# Radiation and PD-(L)1 treatment combinations: immune response and dose optimization via a predictive systems model

**DOI:** 10.1186/s40425-018-0327-9

**Published:** 2018-02-27

**Authors:** Yuri Kosinsky, Simon J. Dovedi, Kirill Peskov, Veronika Voronova, Lulu Chu, Helen Tomkinson, Nidal Al-Huniti, Donald R. Stanski, Gabriel Helmlinger

**Affiliations:** 1 M&S Decisions, Moscow, Russian Federation; 2 Oncology Research, MedImmune, Cambridge, UK; 3 Early Clinical Development, IMED Biotech Unit, AstraZeneca, 35 Gatehouse Drive, Waltham, MA 02451 USA; 4 Early Clinical Development, IMED Biotech Unit, AstraZeneca, Cambridge, UK; 5 Early Clinical Development, IMED Biotech Unit, AstraZeneca, Gaithersburg, MD USA

**Keywords:** Radiation therapy, Immuno-oncology (IO), Checkpoint inhibitors, PD-1, PD-L1, Quantitative systems pharmacology, CT26 tumors, Cancer immunity cycle, Immuno-activation, Immuno-suppression, Dose sequencing and scheduling

## Abstract

**Background:**

Numerous oncology combination therapies involving modulators of the cancer immune cycle are being developed, yet quantitative simulation models predictive of outcome are lacking. We here present a model-based analysis of tumor size dynamics and immune markers, which integrates experimental data from multiple studies and provides a validated simulation framework predictive of biomarkers and anti-tumor response rates, for untested dosing sequences and schedules of combined radiation (RT) and anti PD-(L)1 therapies.

**Methods:**

A quantitative systems pharmacology model, which includes key elements of the cancer immunity cycle and the tumor microenvironment, tumor growth, as well as dose-exposure-target modulation features, was developed to reproduce experimental data of CT26 tumor size dynamics upon administration of RT and/or a pharmacological IO treatment such as an anti-PD-L1 agent. Variability in individual tumor size dynamics was taken into account using a mixed-effects model at the level of tumor-infiltrating T cell influx.

**Results:**

The model allowed for a detailed quantitative understanding of the synergistic kinetic effects underlying immune cell interactions as linked to tumor size modulation, under these treatments. The model showed that the ability of T cells to infiltrate tumor tissue is a primary determinant of variability in individual tumor size dynamics and tumor response. The model was further used as an in silico evaluation tool to quantitatively predict, prospectively, untested treatment combination schedules and sequences. We demonstrate that anti-PD-L1 administration prior to, or concurrently with RT reveal further synergistic effects, which, according to the model, may materialize due to more favorable dynamics between RT-induced immuno-modulation and reduced immuno-suppression of T cells through anti-PD-L1.

**Conclusions:**

This study provides quantitative mechanistic explanations of the links between RT and anti-tumor immune responses, and describes how optimized combinations and schedules of immunomodulation and radiation may tip the immune balance in favor of the host, sufficiently to lead to tumor shrinkage or rejection.

**Electronic supplementary material:**

The online version of this article (10.1186/s40425-018-0327-9) contains supplementary material, which is available to authorized users.

## Background

Radiation therapy (RT) has been used for more than a century and remains an effective treatment for local tumor control in the management of solid malignancies, with up to 50–60% of all cancer patients receiving such treatment [1]. Emerging evidence suggests that RT, in addition to its direct tumor cytotoxic effects, also stimulates specific immune responses which may play an important role in the overall process of RT-induced anti-tumor effects [2]. RT may indeed lead to immunogenic cell death (ICD), which is characterized by the release of damage-associated molecular patterns (DAMPs; e.g.*,* ATP, HMGB-1) from cancer cells, translocation of calreticulin molecules to the plasma membrane, and activation of the cGAS-STING pathway [3]. Together, these factors can facilitate the recruitment and activation of antigen presenting cells (APCs), such as dendritic cells (DCs), to prime tumor antigen specific T cells [4–6]. The lack of a durable immune response to RT in established tumors is thought to be a consequence of an immuno-suppressive tumor microenvironment (TME), which may contribute to disease recurrence and progression. One of the possible suppression mechanisms used by cancer cells to escape immune responses relates to adaptive immune tolerance [7]. In fact, immune checkpoint programmed death 1 receptor (PD-1) and its ligand (PD-L1) are often activated in various cancers and play an important role in inhibiting cytotoxic T cell function [8, 9]. Therapeutic blockade of PD-1 or PD-L1 using monoclonal antibodies (mAbs) have demonstrated encouraging responses in patients with melanoma, non-small cell lung cancer (NSCLC), as well as renal cell and bladder cell carcinoma [10].

Two independent studies established that RT leads to PD-L1 up-regulation on tumor cells, in a variety of experimental syngeneic models [11, 12]. In such preclinical studies, interferon-ɣ (IFNɣ) produced by CD8+ T cells was responsible for mediating tumor cell PD-L1 upregulation after fractionated RT [12]. These studies demonstrated that combined therapy generates robust CD8+ T cell responses, which may improve local tumor control, survival, and protect against tumor growth re-challenge. Importantly, the dosing schedule is critical for synergistic effects of combination therapy to manifest themselves: anti-PD-1/L1 treatment concurrent to RT resulted in higher rates of tumor responses [12, 13].

Despite these encouraging results, there still are gaps in our quantitative understanding of the mechanistic details that underlie the observed synergistic effects. Several parameters are known to be of importance, to maximize RT and immuno-oncology (IO) agent combination synergies, including dose and fractionation of RT, as well as the combination schedule [14]. These, in turn, highly complicate the design of clinical trials and interpretation of clinical outcomes and point to a pressing need for a more quantitatively informed rationale when combining RT and IO therapies, based on a contextual molecular understanding of radiobiology, immune cell dynamics, and tumor microenvironment changes [15] .

Here, we present a quantitative systems pharmacology (QSP) model describing the cancer immunity cycle, with the integration of two therapeutic interventions, RT and PD-(L)1 blockade. In particular, the QSP model captures key kinetic features of immune and tumor cell interactions in mouse CT26 tumors, to characterize tumor size dynamics under vehicle, RT alone, anti-PD-L1 mAb alone, and the combination of both treatments, under various dose regimens and sequencing. In addition, the model takes into account inter-animal variability (IAV); it is therefore able to describe anti-tumor responses at both individual- and population-levels, for all treatments specified above. It further showed that the ability of effector T lymphocytes to infiltrate the tumor in response to systemic antigen exposure is a primary determinant of variability in individual tumor size dynamics in syngeneic CT26 mice. In addition, the model was validated against independent experimental data under CD8^+^ T cell depletion conditions, which resulted in much reduced anti-tumor immune responses, suggesting a critical role for effector T cells in RT-induced tumor shrinkage. Using such a validated QSP model, we gained a deeper understanding of RT + anti-PD-L1 synergistic effects, whereby particular kinetics in the modulation of the local tumor microenvironment result in effective tumor growth inhibition. Furthermore, we show the potential in using this QSP model as an in silico evaluation tool to explore different combination regimens, including various RT doses and fractionation schedules, and in various sequencing with PD-L1 blockade, in order to maximize anti-tumor responses.

## Methods

### Experimental data used for model development

Model development consisted of four main steps, with each step based on a different experimental dataset published by [12, 13].

In a first step, the QSP model was calibrated using experimental data on tumor size dynamics in CT26 syngeneic mice [12]. These data came from a total of 59 BALBc mice, 7 to 16 animals per treatment group, injected with 5 × 10^5^ CT26 cells on Day 0. Animals were then randomly assigned to one of the following treatment arms: (1) control isotype mAb; (2) anti-PD-L1 on Day 7; (3) radiation therapy (RT) with a fractionated dose of 5 × 2 Gy, Days 7 to 11, along with an isotype control mAb; (4) combination treatment with a concurrent schedule of RT (5 × 2 Gy fractionated dose, Days 7 to 11) and anti-PD-L1 mAb on Day 7; (5) combination treatment with sequenced Schedule 1: RT (5 × 2 Gy fractionated dose, Days 7 to 11) and anti-PD-L1 on Day 12; (6) combination treatment with sequenced Schedule 2: RT (5 × 2 Gy fractionated dose, Days 7 to 11) and anti-PD-L1 on Day 19. RT was performed 7 to 11 days after tumor cell inoculation, when tumors reached a minimum size of 100 mm^3^, as described previously [12]. Administration of an anti-PD-1, anti-PD-L1, or isotype control mAb was initiated on Day 1 of the fractionated RT cycle (unless otherwise stated), intraperitoneally (i.p.) 3qw for up to 3 weeks at a dose of 10 mg/kg, in a dose volume of 100 mL/10 g in PBS.

In a second step, model validation was performed using data from CT26 tumor-bearing mice treated with either RT only (7 Gy or 3 daily fractions of 4 Gy), or in combination with an anti-PD-1 mAb dosed at 10 mg/kg 3qw [13]. In this experimental setting, mice were inoculated sub-cutaneously (s.c.) with 5 × 10^5^ CT26 cells. RT was performed 7–10 days after tumor cell injection, when tumors were at least 100 mm^3^ in size, using a Pantak HF-320320 kV x-ray unit (Gulmay Medical, U.K.). The machine was operated at 300 kV, 9.2 mA, with filtration fitted in the x-ray beam to give a radiation quality of 2.3 mm Cu half-value layer. Mice were positioned at a distance of 350 mm from the x-ray focus, where the dose rate was 0.80 Gy/min and treated using tangential beam delivery. Administration of anti-PD-1 (clone RMP1–14), anti-PD-L1 (clone 10F.9G2) or isotype control mAb (Biolegend, U.K.) started on Day 1 of the fractionated RT cycle via i.p. injection 3qw for 1 week at a dose of 10 mg/kg, in a dose volume of 100 μl / 10 g in PBS.

In a third step, model validation was performed using data from CT26 tumor-bearing mice treated with either a fractionated RT and anti-PD-L1 mAb combination, or with an anti-CD8 mAb added to that combination [12]. All other experimental conditions were the same as the ones described under the first step.

In a fourth step, data on PD-L1 expression in CT-26 cells were used for further model validation [13].

### Mathematical modeling of the cancer immunity cycle, with the incorporation of RT and anti-PD-L1 therapies

**Fig. 1:**
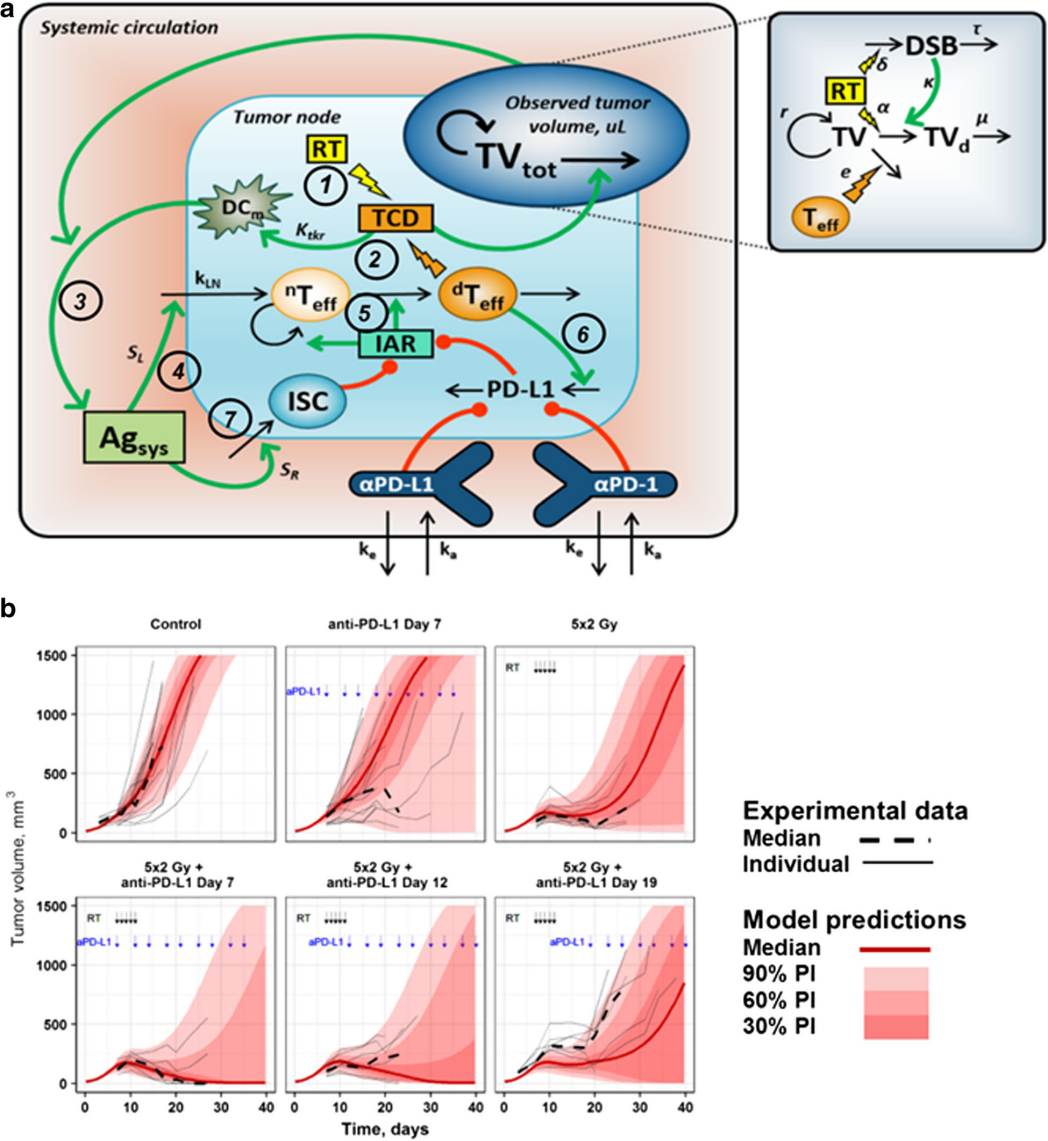
**a** Structural elements and interactions captured in the IO QSP model. Abbreviations used: *TCD*: tumor cell death rate function; *DC*_*m*_: level of mature DCs; *IAR*: immune activation rate; *Ag*_*sys*_: systemic level of tumor antigen; *PD-L1*, PD-L1 immuno-suppressive component; *ISC*: immuno-suppression cells; ^*n*^*T*_*eff*_: non-differentiated T cells; ^*d*^*T*_*eff*_: cytotoxic effector T cells; *IAR*, immune activation rate*; DSB*: double-strand breaks; *TV*_*tot*_ *= TV + TV*_*d*_, where *TV* and *TV*_*d*_ are volumes of, respectively, proliferating cells and radiation-damaged non-proliferating tumor cells. **b** Distributions of population model predictions and corresponding tumor dynamics data. Black arrows: RT administration (fractionated dose of 5 × 2 Gy); blue arrows: anti-PD-L1 mAB administration (3qw for 3 weeks). Values on plots indicate number of complete tumor rejections and number of animals in the experiment. Experimental data are taken from Dovedi et al., 2014. [12]

We developed the mathematics of the QSP model, which includes key elements of the cancer immunity cycle and the tumor microenvironment [16], tumor growth, as well as dose-exposure-target modulation features, to reproduce experimental data of CT26 tumor size dynamics upon administration of RT and/or a pharmacological IO treatment such as an anti-PD-L1 agent (Fig. 1a). Ordinary differential equations were implemented to describe and simulate proliferation and differentiation of effector T cells, tumor cell kill processes, and PD-L1 expression dynamics (see Section S2 in Additional file 1). To describe the development of an immune response within the model, we incorporated critical players such as cytotoxic effector T lymphocytes (^*d*^*T*_*eff*_) and their non-differentiated precursors (^*n*^*T*_*eff*_), immuno-suppressive cells (*ISC*), mature dendritic cells (*DC*_*m*_), a systemic level of tumor antigen presentation (*Ag*_*sys*_), a PD-L1 expression level and its negative feedback to facilitate the process of ^*d*^*T*_*eff*_ exhaustion and apoptosis. Figure 1a schematically depicts all structural elements of the QSP model. Radiation-induced tumor cell death (*TCD*) (Step 1), initiates an immune response by stimulating DC maturation (Step 2), which subsequently facilitates *Ag*_*sys*_ (Step 3). The build-up of *Ag*_*sys*_ in concert with DC maturation leads to the recruitment of tumor-infiltrating T cells (Step 4) and their respective proliferation and differentiation (Step 5), which then induces an up-regulation of PD-L1 expression levels (Step 6). Also, elevated levels of *Ag*_*sys*_ gradually facilitate the accumulation of different *ISC*, including tumor antigen-specific regulatory T cells in the tumor (Step 7). This, in turn, contributes to an up-regulation of immuno-suppression and, together with overall levels of PD-L1, determines the *Immune Activation Rate* (IAR). RT effects on tumor cell kill and the pharmacokinetics and pharmacodynamics of anti-PD1/L1 mAbs were also characterized in the model.

The model was calibrated based on CT26 tumor size data in individual animals using a nonlinear mixed-effects modeling technique; model quality was evaluated using multiple criteria (Fig. 1b, Additional file 1: Figure S1). All model parameters are summarized in Table 1. To incorporate IAV in responses observed in the tumor size data, we considered a parameter, *S*_*L*_, which reflects the ability of T cells to infiltrate tumor tissue under *Ag*_*sys*_ presentation, and whose values could be varied via a random effect to describe IAV and thereby achieve the best model fitting to tumor size dynamics (Additional file 1: Figure S2) and percentage of ‘responders’ (Additional file 1: Figure S3). Interestingly, the exponential parameter, *r*, which describes the intrinsic tumor growth rate, showed the highest sensitivity score among all model parameters (Additional file 1: Figure S4), meaning that even small changes in this parameter value may significantly affect overall efficacy, for a given treatment. However, adding a random effect to this parameter did not help in adequately describing the available tumor size data, thus changes in the intrinsic tumor growth rate could not well explain the observed IAV in tumor dynamics, across animals and within a given treatment. In contrast, even a small-to-moderate variability in *S*_*L*_ (Additional file 1: Figure S1C) provided a much-improved fit to the tumor size data, as shown by the respective differences in objective function values (Additional file 1: Table S1). Further details of model development and evaluation can be found in Additional files.

**Table 1:** Model parameters. (RSE, Relative standard error)

Parameter	Unit	Description	Value	RSE (%)	Comments and references
r	d^−1^	Tumor growth rate	0.4		Taken from [41]
TV_max_	μL	Maximal size of tumor	2500		Taken from [41]
d_0_	d^− 1^	Spontaneous death rate of tumor cells	0.01		Assumed and to preserve d_0_ < < r, given the proportion of apoptotic vs. proliferating cells is minor, in growing syngeneic tumors [25].
k_LN_	cells/d	Maximal influx rate of ^*n*^*T*_*eff*_ cells	279	8	Estimated based on tumor growth data
S_L_	n/a	T cell ability to infiltrate tumor tissue under systemic antigen exposure	8.89	13	Estimated based on tumor growth data
Ω_SL_	n/a	Random effects on *k*_*LN*_	0.696	10	Estimated based on tumor growth data
k_pro_	d^−1^	^*n*^*T*_*eff*_ proliferation rate constant	3.0		Estimated based on a minimal duration (6 h) of the cell division cycle [42]
k_dif_	d^−1^	^*n*^*T*_*eff*_ differentiation rate constant	3.2		Assumed, to preserve observed ^n^T_eff_/^d^T_eff_ ratio in tumor tissue [43]
k_el_	d^−1^	^*n*^*T*_*eff*_ elimination rate constant	0.2		Estimated based on half-life of primed T cells [42]
k_apo_	d^−1^	^*d*^*T*_*eff*_ apoptosis rate constant	2.0		Estimated based on activated cytotoxic T cells in tissue [42]
e	d^− 1^**•** cell^− 1^	Rate of tumor cell kill by ^*d*^*T*_*eff*_	0.001		Assumed based on CD8^+^ cell density in CT26, controlling tumor regrowth after RT [12, 25]
K_pdl_	cells	Sensitivity of PD-L1 expression up-regulation to ^*d*^*T*_*eff*_ count	478	23	Estimated based on tumor growth data
k_pdl_	d^−1^	PD-L1 up-regulation rate constant	1.0		PD-L1 response was assumed to reach a steady-state in about 1 day, as shown in vitro [44]
K_tcd_	d^− 1^	Sensitivity of *DC*_*m*_ to *TCD* value	0.2		Assumed to be sufficiently high to stimulate DC maturation in the TME [17]
S_R_	n/a	Sensitivity of cellular immuno-suppression to accumulation to systemic Ag level	30.5	12	Estimated based on tumor growth data
α	Gy^− 1^	Linear component of radiation effect	0.146	9	Estimated based on tumor growth data
δ	Gy^−1^	*DSB* formation per cell per Gy	19		Taken from [45]
τ	d (day)	*DSB* repair time	0.02		Taken from [46]
μ	d^−1^	Elimination rate of radiation-damaged tumor cells	0.1725		Calculated from the half-life value [47]
V_d_	L	Volume of distribution for PD-L1 mAb *i.p.* PKPD model in mouse	0.003		Estimated from [48]
k_a_	d^−1^	*i.p.* absorption rate	8.0		Estimated from [48]
kel_mAB_	d^−1^	mAb elimination rate	0.15		Estimated from [48]
K_D_	nM	mAb PD-L1(PD-1) binding affinity	30		Taken from internal data
a	μL	Constant component of residual error	21.2	13	Estimated based on tumor growth data
b	n/a	Proportional component of residual error	0.176	10	Estimated based on tumor growth data

Methodological details on the proposed structure of the QSP mathematical model and corresponding model development, analysis, and diagnostics approaches are fully described in the Additional files document.

### Software

Model development and analyses were performed using the IQM systems pharmacology and pharmacometrics toolbox (IntiQuan, Basel, Switzerland), based on MATLAB® 2013b (The Mathworks®, Natick, MA, USA). Visualization of model simulations was performed in the R software version 3.2.5, using the ggplot2 2.1.0 and plot3D 1.1 packages. Nonlinear mixed-effects (NLME) model analysis, parameter and relative standard error estimation were based on the stochastic approximation expectation maximization (SAEM) algorithm and performed using the Monolix® software (Lixoft, Antony, France).

## Results

### Model validation against newly, independently generated sets of experimental tumor size data

**Fig. 2:**
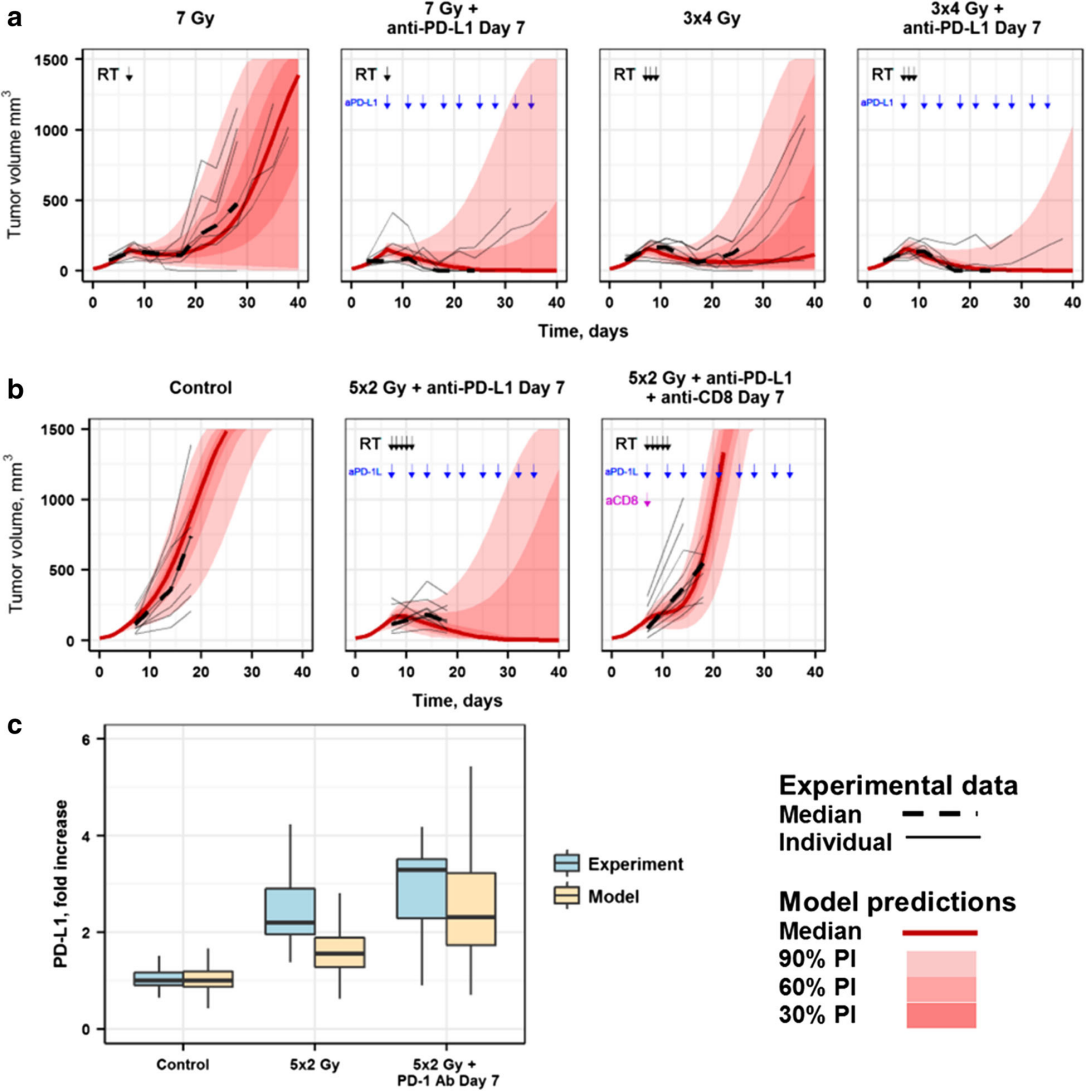
**a** Tumor size dynamics data and model predictions. Experimental data from Dovedi et al., 2017 [13]. **b** Tumor effects following depletion of CD8^+^ T cells. Experimental data from Dovedi et al., 2014 [12]. Black arrows: RT administration; blue arrows: anti-PD-L1 mAb administration. **c** Population model predictions of PD-L1 expression level dynamics, and corresponding measurements of PD-L1 MFI (mean fluorescence intensities) on tumor cells (CD45^−^). Comparisons of PD-L1 expression levels measured at Day 12–18 (blue boxes or dots) in experiments vs. corresponding model-based simulations (beige boxes). All values were normalized to PD-L1 absolute median values from the control group. Experimental data from Dovedi et al., 2017 [13]

The predictive power of the QSP model was assessed via an external cross validation: the model was used in a forward-simulation mode, by simulating new experimental scenarios for which tumor size data had been independently generated, to indeed determine whether we could predict such data – data which had not been used in the model development and evaluation steps described above. The following scenarios were simulated for this purpose, with a post-hoc verification against the existing data [12, 13]: (1) a single 7 Gy dose RT alone and in combination with anti-PD-L1 (10 mg/kg 3qw); (2) fractionated 3 × 4 Gy RT alone and in combination with anti-PD-L1 (10 mg/kg 3qw); (3) combination therapy of fractionated 5 × 2 Gy RT and anti-PD-L1 treatment (10 mg/kg 3qw), with and without administration of an anti-CD8 antibody; (4) PD-L1 expression levels upon RT alone (5 × 2 Gy) and RT + anti-PD-1 combination. The QSP model adequately reproduced all these additional experimental data (Fig. 2), demonstrating its ability to predict individual tumor responses to de novo mono- and combination treatment regimens, and to provide, simultaneously, mechanistic insights of the underlying molecular and cellular dynamical interplays in tumor tissue such as ^*d*^*T*_*eff*_, immuno-suppressive forces, levels of PD-L1 expression. Further details on model validation can be found in Section S5 in Additional file 1.

### Mechanistic rationale of RT and anti-PD-L1 synergistic effects

**Fig. 3:**
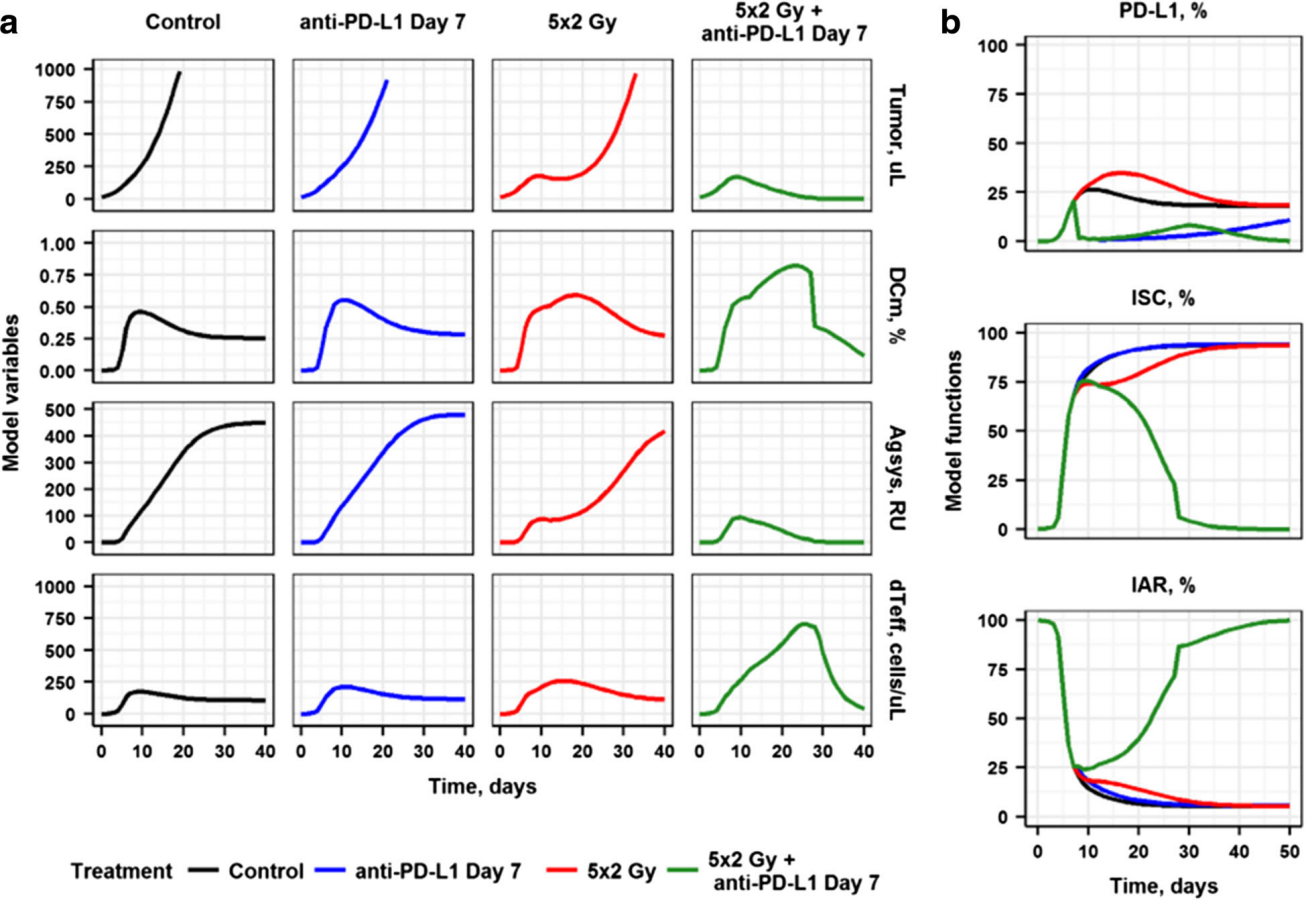
Model-based predictions illustrating the dynamic interplay among key cellular and molecular players in the cancer immunity cycle. **a** Simulations of model variables: tumor size dynamics, expressed as tumor volume over time; *DC*_*m*_; *Ag*_*sys*_; and ^*d*^*T*_*eff*_. **b** Model driving functions: *PD-L1*; *ISC*; and *IAR*. All treatments start at Day 7 after tumor implantation

Detailed longitudinal data reflecting time-dependent changes of molecular and cellular signals in the TME are difficult to obtain, often due to experimental and cost limitations. Model-based explorations of immuno-suppressive and -activating drivers may instead provide a deeper understanding of the interplay among key players in the cancer immunity cycle and the regulation of anti-tumor responses under various treatment scenarios. The QSP model may thus be used to follow, via kinetic and quantitative simulations, key model variables which represent actual biology or can be transformed to give actual biological meanings, including tumor size (expressed as tumor volume over time), levels of *DC*_*m*_, *Ag*_*sys*_, ^*d*^*T*_*eff*_, as well as immuno-suppressive (*PD-L1* and *ISC*) and immuno-activating (*IAR*) driving functions, under any desired treatment conditions: control, RT and anti-PD-L1 monotherapies, and combination (Fig. 3).

Tumor size exhibited an exponential growth behavior under control conditions, as expected, even though a transient T cell immune activation - caused by an accumulation of *Ag*_*sys*_ - was observed (Fig. 3a). The transient immune activation was abrogated by an immune suppression which develops within the lumped *IS*C function. As a result of increasing *ISC* and rising PD-L1 levels, the *IAR* function was greatly reduced to a low level of 5% by Day 20 (Fig. 3b), resulting in subsequent rapid tumor growth (Fig. 3a). Consistent with data from other syngeneic tumor models [11, 12], anti-PD-L1 treatment alone did not effectively inhibit tumor growth. Moreover, only a modest increase in ^*d*^*T*_*eff*_ could be observed (Fig. 3a). These results indicate that the role of PD-L1-related immune tolerance may be secondary and provide only a maximal ~ 25% effect of the overall immuno-suppression level (Fig. 3b). In contrast, tumor cells eliminated via RT resulted in a more direct delay in tumor growth. Such rapid cell death may indeed provide a transient increase in *TCD*, which may then facilitate the DC maturation process – in concordance with the concept of *ICD* and related DAMP-initiated molecular events [17]. In fact, the QSP model supported this view, showing improved *Ag*_*sys*_ and mildly-increased T cell priming and influx into tumor tissue, under RT (Fig. 3a). However, the RT-dependent immune response is transient. Thus, on Days 15 to 20, RT effects on tumor dynamics became much smaller, with tumors presenting re-growth, thus leading to increases in *Ag*_*sys*_ and *ISC* accumulation, but also decreases in *DC*_*m*_ levels, resulting in poor anti-tumor responses at such points in time (Fig. 3a). It should also be noted that model-based time-course predictions of PD-L1 expression levels were transient for control and RT treatment conditions, thus pointing to a critical time window to possibly inhibit PD-L1-related immuno-suppression efficiently.

Administration of an anti-PD-(L)1 mAb concurrently with RT resulted in a dramatic increase in tumor growth inhibition, with a majority of animals exhibiting complete tumor rejection. Under such a treatment scenario, a sufficient increase in *DC*_*m*_ levels as well as a gradual activation and accumulation of ^*d*^*T*_*eff*_ in tumor tissue occur. Interestingly, levels of *Ag*_*sys*_ were typically lower vs. other experimental conditions, because of smaller tumor volumes (Fig. 3a). This indicates that antigen exposure, while certainly necessary, may not be entirely sufficient to generate an effective immune response. Hence, TME conditions that prevail following RT and more rapid, higher *DC*_*m*_ levels are crucial to achieve an overall anti-tumor effect. Another interesting observation is that boosting of an immune response via higher *DC*_*m*_ levels – and with relatively modest increases in *Ag*_*sys*_ – can lead to an effective ^*d*^*T*_*eff*_ response, without an overwhelming accumulation of *ISC* (Fig. 3b). Once the PD-1/PD-L1 axis is sufficiently inhibited through a blocking antibody, rapid RT-associated ^*n*^*T*_*eff*_ influx is no longer suppressed (as observed under RT monotherapy), and active ^*d*^*T*_*eff*_ may build up in tumor tissue and exert their inhibitory effects on tumor growth (Fig. 3a). Due to these collective effects, the modeled *IAR* level was restored up to a full value of 100% corresponding to the prevalence of the complete responders in the treatment group. Therefore, the higher efficacy that resulted from a concurrent administration of RT with an anti-PD-L1/PD-1 antibody can be explained by a favorable dynamic interplay between a rapid boosting of T cell influx and a simultaneous initial activation of cytolytic capacity of these cells in the tumor.

### Factors driving anti-tumor response and tumor rejection

Tumor growth dynamics in syngeneic mouse models are known to show high *IAV*, with a very broad range in the observed slopes of tumor volume increases, even under control conditions or under various treatment scenarios (Fig. 1b). An additional challenge in the quantitative analysis of such data lies in the relatively small number of animals used per treatment cohort. Hence, cohort averages (e.g., of tumor size, as mean or median values) may not be maximally informative in the assessment of treatment efficacy and of related biomarker behaviors. One way to assess efficacy more comprehensively would be to look at individual animal responses, and define a percentage of ‘responders’ based on animals showing complete tumor response (see Section S6 in Additional file 1).

**Fig. 4:**
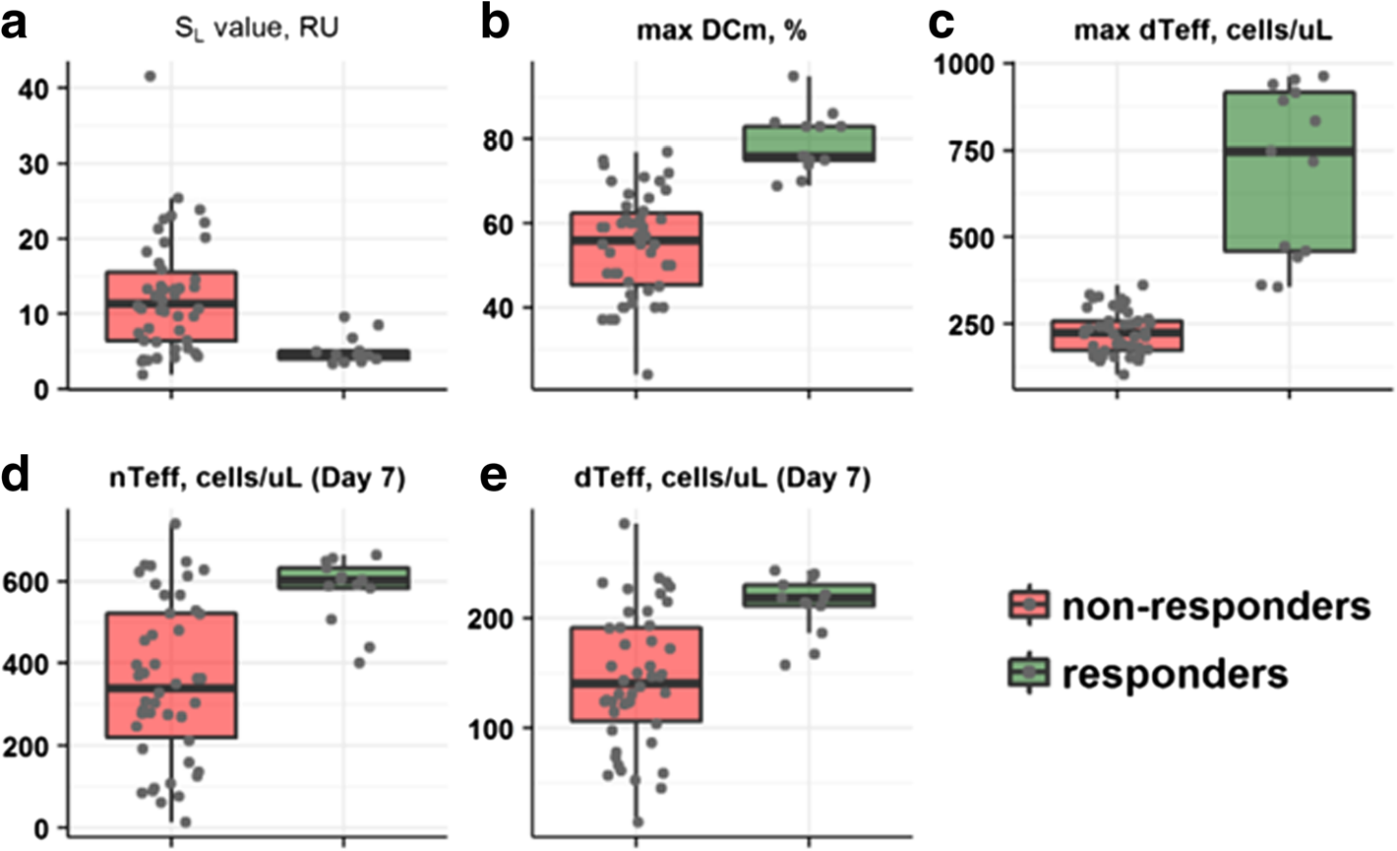
Mechanistic differences between animals with progressing tumor growth (‘non-responders’, orange color) vs. animals with full efficacy (tumor rejection – ‘responders’, green color). **a** Distribution of individual *S*_*L*_ parameter values. **b** Maximal *DC*_*m*_ levels. **c** Maximal counts of intra-tumoral ^*d*^*T*_*eff*_ cells. **d** Intra-tumoral ^*n*^*T*_*eff*_ cells before treatment start. **e** Intra-tumoral ^*d*^*T*_*eff*_ cells before treatment start. Grey dots: individual parameter values or model simulations, respectively

The QSP model was used in such context to better understand underlying mechanisms of tumor response in individual animals. For example, individuals with tumor rejection showed smaller *S*_*L*_ values vs. individuals with progressive tumor growth (Fig. 4a). Based on these observations, one may conclude that higher and more rapid T cell infiltration in such individuals drove anti-tumor efficacy. This model-based finding was supported further by simulations, e.g., animals with tumor rejections indeed exhibited higher maximal *DC*_*m*_ levels (Fig. 4b) and correspondingly higher intra-tumor ^*d*^*T*_*eff*_ counts (Fig. 4c). Interestingly, the before-treatment counts of both ^*n*^*T*_*eff*_ and ^*d*^*T*_*eff*_ were significantly higher for the animals experiencing complete response, providing additional evidence of the predictive value of tumor lymphocyte infiltration (Fig. 4d, e).

**Fig. 5:**
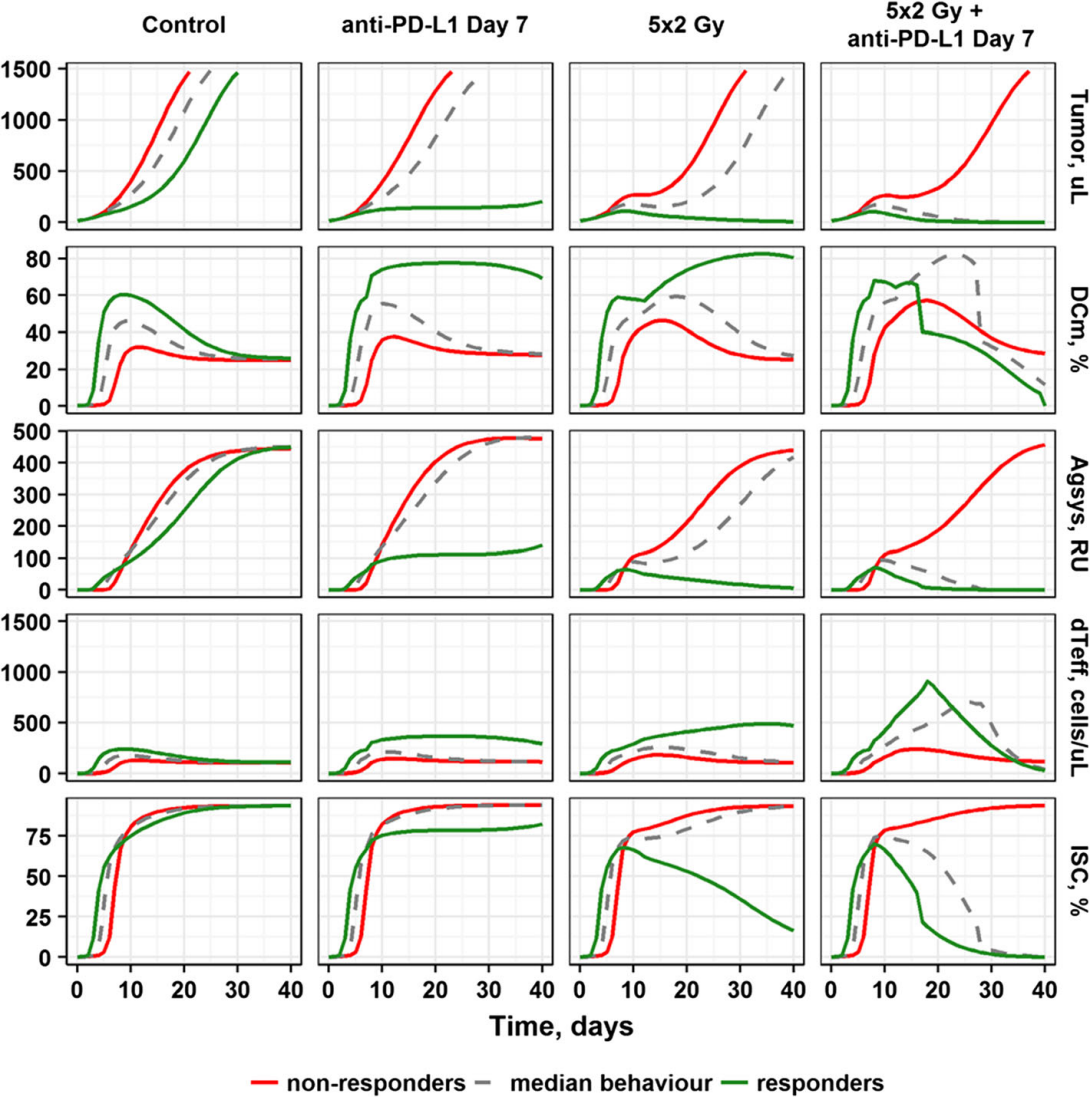
Model-based simulations predicting mechanistic features distinguishing ‘responders’ (animals exhibiting tumor rejection; green lines) vs. ‘non-responders’ (animals exhibiting, ultimately, tumor progression; red lines). Green and red simulation curves correspond, respectively, to individuals with *S*_*L*_ values of *1.77* and *22.63* RU, which represent the 10th and 90th percentiles of the *S*_*L*_ parameter value distribution. Dashed grey lines correspond to typical individuals with an *S*_*L*_ value of *6.95* (the median of the *S*_*L*_ parameter value distribution). *DC*_*m*_; *Ag*_*sys*_; ^*d*^*T*_*eff*_; *ISC* levels in tumor. All treatments started at Day 7 *post* tumor implantation

To further explore mechanisms underlying variability in the observed responses to treatments, we simulated tumor size dynamics, as well as *DC*_*m*_, *Ag*_*sys*_, activated intra-tumor ^*d*^*T*_*eff*_ counts, and PD-L1 expression levels for two extreme cases corresponding to individuals within either the 10th or 90th percentile of *S*_*L*_ values (T cell effectiveness in infiltrating tumor tissue following *Ag*_*sys*_ exposure) of, respectively, 1.77 and 22.63 RU. In accordance with the tumor response patterns shown above, such lower and upper *S*_*L*_ values could be mapped, respectively, to non-responders (tumor progression) or complete responders (tumor rejection) following therapeutic interventions (Fig. 5). Under control conditions, where the probability of a tumor response is very low, extreme values in *S*_*L*_ simply resulted in different slopes (rates) of tumor growth. Under all treatment scenarios simulated, a key driver of a complete tumor response is a rapid (e.g., during the first week following start of treatment) and robust accumulation of activated ^*d*^*T*_*eff*_ cells in tumor tissue. This intense ^*d*^*T*_*eff*_ infiltration is in fact initiated and maintained by higher levels of *DC*_*m*_ (Fig. 5).These results provide a mechanistic rationale underlying variability in CT26 tumor growth dynamics and in their responses to RT and anti-PD-L1 treatments, and demonstrate that an early onset and effective ^*n*^*T*_*eff*_ infiltration, supported by higher and sufficiently sustained levels of DC_m,_ are necessary to overcome the development of immuno-suppression within the TME and to ultimately achieve tumor rejection.

### RT and anti-PD-L1 combination therapies: Optimization of dose schedules and sequences

**Fig. 6:**
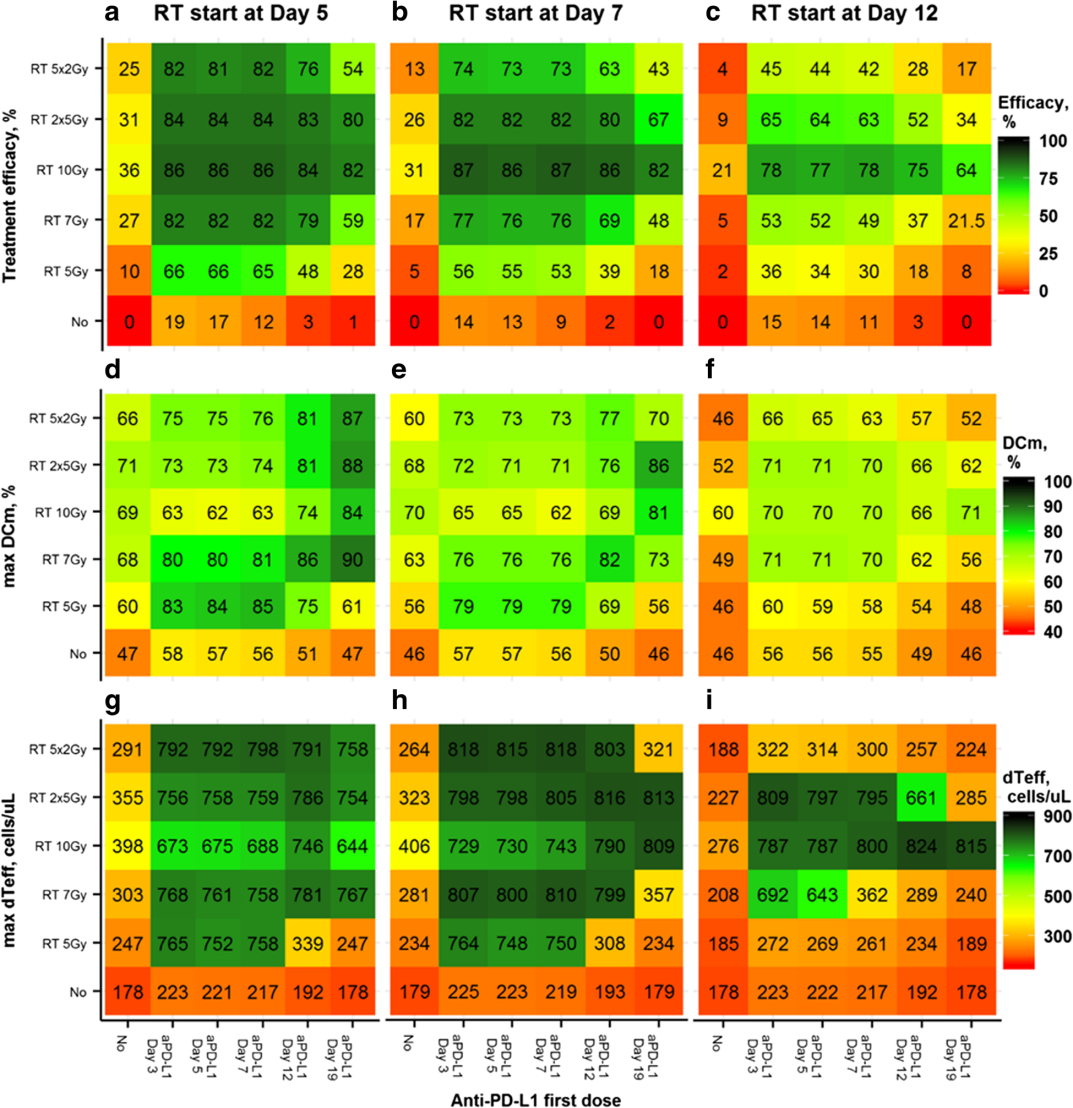
Model simulations of various dose scheduling and sequencing in RT + anti-PD-L1 combination therapies. Panels **a**-**c** Efficacy simulation results, summarized as percentages of ‘responders’ (animals exhibiting full tumour rejection; defined as a total tumor volume ≤ 10 mm^3^ on Day 50 following treatment start), median (values in brackets), based on 1000 virtual studies with 100 animals per study, respective 90% CI are shown in Additional file 1: Table S2. Panels **d**-**f** Simulations of corresponding maximal *DC*_*m*_ level. Panels **g**-**i** Simulations of corresponding ^*d*^*T*_*eff*_. Panels **a**, **d**, **g** – RT started on Day 5 after injection of tumor cells; Panels **b**, **e**, **h** – RT started on Day 7 after tumor cell injection; Panels **c**, **f**, **i** – RT started on Day 12 after tumor cell injection. Confidence intervals are provided in Additional file 1:Table S2

Having elucidated the early dynamics necessary for effective tumor growth inhibition, we sought to determine optimal combination regimens of RT + anti-PD-L1 treatments in CT26 tumors, through model-based simulations. Thus, we used the QSP model to simulate a wide spectrum of realistic treatment scenarios. In particular, five different dosing times of anti-PD-L1 mAb administration (Day 3, 5, 7, 12, or 19 after tumor cell injection) were simulated, in combination with several RT regimens. Different RT regimens and different times of treatment start were also investigated, including conventional fractionation (10 Gy delivered in 5 daily fractions of 2 Gy), hypo-fractionation (10 Gy delivered in 2 fractions), and single doses (5, 7 and 10 Gy) on Day 5, 7, or 12 (Fig. 6a-c). All simulation results were summarized as percentages of ‘responders’, which may be interpreted as a ‘complete response rate’, while also taking into account uncertainty in parameter estimates and *IAV*. We also simulated and reported maximal levels of *DC*_*m*_ (Fig. 6d-f), and ^*d*^*T*_*eff*_ counts (Fig. 6g-i) accumulated in the tumor compartment, for all scenarios.

Model simulations show that monotherapies of either RT or anti-PD-L1, in general, resulted in none-to-modest response rates (0% to 27%), consistent with experimental data [11, 12]. In both sets of monotherapy scenarios, *DC*_*m*_ could, at times, increase, although this did not translate into intra-tumoral ^*d*^*T*_*eff*_ accumulation and subsequent tumor rejection. In contrast, the various combination scenarios which were simulated led to significantly higher response rates, typically in the 40% to 70% range. In these more efficacious combination scenarios, intra-tumoral ^*d*^*T*_*eff*_ counts were significantly higher while not entirely reflecting simulated *DC*_*m*_ levels. Interestingly, the highest response rates were obtained for combination scenarios where an anti-PD-L1 mAb was administered prior to, or concurrent with RT. Sequential scheduling of anti-PD-L1 treatment administered after RT revealed decreased response rates which were progressively poorer with longer intervals between RT and anti-PD-L1 mAb administration (Fig. 6a-c).

Simulations of combinations also showed that response rates were significantly dependent upon times of treatment start following tumor implantation; a result which may be explained by the critical roles of initial tumor size and levels of TME immuno-suppression at baseline. Thus, combination scenarios with a single high dose of RT as late as Day 12 after tumor implantation resulted in high response rates (Fig. 6c), with prior or concurrent anti-PD-L1 administration. However, in scenarios with treatment starting 19 days after tumor implantation, all tested combination schedules revealed modest efficacy results and an almost complete lack of RT-induced immune modulation (Additional file 1: Figure S5).

Conventional fractionated and hyper-fractionated RT regimens, if administered earlier in the combination setting and to less-established tumors (e.g., Day 5 or 7 after tumor inoculation), resulted in response rates nearly as high as the high single dose of RT. However, these fractionated regimens were significantly less effective in more established tumors (e.g., if treatment was initiated at Day 12 post tumor implantation; Fig. 6a-c). At the same time, fractionated RT regimens in the combination setting do result in high levels of *DC*_*m*_ (Fig. 6d-f), and show higher overall effects on the system’s immunity.

In summary, anti-PD-L1 administration prior to, or concurrently with RT revealed more synergistic effects, which presumably materialized through more favorable dynamics between RT-induced immuno-modulation and reduced immuno-suppression of T cells through anti-PD-L1.

## Discussion

Modeling and simulation methods have a long history of applications in support of preclinical and clinical research, as well as decision-making in Oncology, including pioneering efforts in breast cancer with application of the Gompertz model [18]. These were followed by many other attempts to develop models describing, e.g., different aspects of carcinogenesis, effects of therapeutic interventions [19, 20]. In radiation oncology, modeling has traditionally been used to describe the effects of ionizing radiation damage on tumor and other cell types, starting from a basic LQ formalism [21] and evolving to more realistic approaches, e.g., taking into account tumor heterogeneity [22] or physiologically-based T cell trafficking [23]. However, there is very limited modeling work that takes TME contributions into account – an essential step towards the systematic investigation and understanding of synergies between immuno-therapies and RT [24]. In fact, none of the models focusing on RT + IO agent combinations have been successfully constrained with experimental data, thereby restricting their predictive ability and use for prospective simulations. The aim of the present research was to derive a parsimonious, fit-for-purpose, yet mechanistically-driven approach to (i) describe essential features underlying the biological complexity of the cancer immunity cycle, and (ii) keep the model quantitatively valid by integrating a sufficient amount of data from multiple in vivo and in vitro sources, while obtaining precise parameter estimates to allow for prospective, predictive simulations. To achieve these goals, we developed a first-in-class QSP model which captures synergistic effects observed between RT and immune checkpoint blockade. The model integrates key steps of the cancer immunity cycle, such as release of tumor antigens and their presentation to DCs, processes of T cell priming, proliferation, differentiation as well as immune cell interaction with tumor cells, and processes of immuno-suppression in the TME. The model was calibrated based on the growth dynamics of CT26 syngeneic tumors under various therapeutic interventions, e.g., administration of RT or an anti-PD-L1 mAb alone, and their combinations. To assess the predictive power of the model, we simulated several experimental scenarios, which allowed us to compare prospective simulations with independently generated experimental data. We showed that the model made accurate predictions at individual and cohort levels, around multiple features such as the evaluation of different RT/anti-PD-L1 dose sequencing and scheduling, as well as the underlying, interactive molecular and cellular marker dynamics leading to tumor size modulation.

A striking finding uncovered during model development is that the observed IAV in tumor size dynamics is caused by individual differences in T cell tumor infiltration properties. Thus, after having applied random effects to various parameters in the model and followed by several model diagnostic techniques, we confirmed the hypothesis that the ability of T cells to infiltrate the tumor following *Ag*_*sys*_ exposure (a random effect applied to the *S*_*L*_ model parameter) can well explain IAV in CT26 tumor dynamics. A model-based sensitivity analysis suggested that other parameters, such as the intrinsic tumor growth, *r*, can significantly influence tumor size modulation, however applying a random effect to *r* did not allow us to reproduce the observed experimental IAV as well as applying it to *S*_*L*_. Such a finding is in good agreement with data obtained under inhibition of T cell tumor infiltration by fingolimod, a sphingosine-1-phosphate receptor modulator, which significantly diminished the IAV in tumor growth patterns under both no treatment and treatment conditions [13]. Additionally, individual differences in the ability of T cells to infiltrate tumor tissue can explain the mechanism of complete tumor response (rejection) under various treatment conditions. As shown in the Results section, animals with a higher T cell ability to infiltrate the tumor can be characterized by higher levels of *DC*_*m*_ and consequently higher counts of ^*d*^*T*_*eff*_ cells, which then may cause improved tumor shrinkage or elimination. In fact, similar results have been reported, experimentally, in TUBO tumor-bearing syngeneic mice: animals with RT-induced tumor rejection or stable disease exhibited higher numbers of active ^*d*^*T*_*eff*_ cells, both in draining lymph nodes and in tumor tissue [25].

Data from experimental studies are, however, typically sparse and not amenable to an exhaustive investigation of the full time course of molecular and cellular biomarkers. Simulations via our mechanistic QSP model does allow for such time course studies, complementing and extrapolating beyond sparse experimental data points, which in turn may help in reconstructing causal effects leading to an outcome (tumor size modulation), in a highly nonlinear, multi-variate dynamical system such as the cancer immune cycle [26]. For example, in the context depicted here, individuals with improved tumor growth inhibition responses also experienced rapid ^*d*^*T*_*eff*_ accumulation following start of treatment, a feature which may favorably counteract the subsequent development of immuno-suppression. These modeling results suggest that a baseline (pre-treatment) marker measuring the ability of effector T cells to infiltrate the tumor may have a predictive, rather than prognostic value in evaluating tumor response [27].

Looking further into these dynamical aspects, we inferred a transient time window of opportunity, with respect to the combination synergy, whereby the balance between ^*d*^*T*_*eff*_ cell infiltration and the development of immuno-suppression (through multiple possible mechanisms) is tilted in favor of the former. A precise estimation of such a time window may help maximizing positive treatment outcome and optimizing clinical trial design [15]. In the modeling framework discussed here, we focused on two immuno-suppression axes, (i) PD-L1-dependent, and (ii) a generalized immuno-suppressive function which combines the effects of regulatory T cells, myeloid-derived suppressor cells (MDSC) and other immuno-suppressive cell types. Although our approach worked well for the quantitative description of CT26 tumor dynamics data, it is possible that for other syngeneic tumor models and/or forms of IO treatment, a more specific description of immuno-suppressive mechanisms may be needed. It has been shown, indeed, that syngeneic experimental models may significantly differ in their TME composition during tumor growth [28]. Further deciphering of immuno-suppressive mechanisms, e.g., the role of different MDSC subsets, tumor-associated macrophage polarization [29–31], will allow for updates of this QSP model and its application to other IO modality combinations.

Another key finding in this study is that anti-PD-(L)1 administration prior to, or concurrently with RT resulted in higher efficacy (tumor responses), vs. anti-PD-(L)1 post RT. Following our immune markers in the model, this can be explained mechanistically by a time window of opportunity, for ^*d*^*T*_*eff*_ to actively accumulate in tumor tissue (as stimulated by a transient RT-induced immune modulation), given a background of temporarily reduced PD-L1 immuno-suppression (thanks to a prior, or concurrent anti-PD-(L)1 administration). The same mechanistic rationale may serve to explain why treatment efficacy decreases with more established, higher-volume tumors at baseline (Fig. 6, moving from 6A to 6C): the more established a tumor, the more substantial the gradual accumulation of immuno-suppressive forces in the TME, and the smaller the window of opportunity for immune activation (through the synergistic mechanisms-of-action of RT + anti-PD-(L)1) to overcome resident immuno-suppressive forces. Such simulation results are in agreement with recent experimental data, which reported on the therapeutic effects of various RT regimens in large, established CT26 and MC38 tumors [32].

While the QSP model adequately captured key T cell mechanisms in the TME, one limiting assumption is that RT would directly affect highly proliferating tumor cells only, and not endothelial or immune cells. This assumption limits the use of the current model to simulations of RT regimens ≤10 Gy; beyond such an RT dose, the linear-quadratic equation used in the model would require a significant adaptation for additional RT-induced effects [33, 34]. For example, high single RT doses (8–16 Gy) are known to induce endothelial cell apoptosis, which may lead to changes in tumor vasculature [35]. In addition, tumor-associated fibroblasts may be affected by RT, which may further impact therapeutic results [33–35]. Furthermore, as shown recently, a single RT dose of 10 Gy and higher can affect T cell infiltration into tumor tissue [13]. Our model, however, can be used for the optimization of combination treatment sequences and schedules, in the range of single- and fractionated doses of RT typically used in a majority of preclinical studies [11, 12]; only a limited number of experimental studies considered alternative, significantly higher RT treatment settings [32].

In this simulation study, we did not find significant differences between fractionated- vs. equivalent high single dose RT regimens, when combined with anti-PD-L1. However, the impact of RT dose fractionation on anti-tumor response also depends on the intrinsic radiosensitivity of tumors; further investigations would be required, to explore dose fractionation effects in depth, using different experimental models of tumors with varying radiosensitivity [22].

Given the large number of ongoing trials combining RT with various PD-(L)1-targeting agents [36], there is a need for a common quantitative framework of reference, to leverage existing preclinical data and to support the design of future trials based on detailed mechanistic understanding and appropriate dosing regimens [2]. Translating IO-based modalities from a preclinical setting into the clinic includes the traditional challenges in translational oncology, e.g., the adequacy of existing experimental models and differences in disease progression. The dynamics of tumor growth in syngeneic mice are fundamentally different from cancer progression in human, due to numerous factors such as tumor heterogeneity, clonal evolution, and host morphology [37]. Despite some limiting assumptions, the quantitative dynamic model described here provides a basis for an in-*silico* evaluation tool that integrates key features of the cancer immunity cycle and may be used for clinical translation. Supportive results, in a most recent retrospective analysis of patients receiving an anti-PD-1 antibody concurrently with brain-directed radiation, showed that such a concurrent combination treatment can lead to a significant increase in median survival, in several cancer indications [38] – a result in good agreement with a series of earlier reports [39, 40]. Based on our model-based analysis, we propose that anti-PD-(L)1 treatment prior to, or concurrently with RT may bring about further clinical benefits vs. RT followed by anti-PD-(L)1. Finally, due to its parsimonious mechanistic nature, our model may be tailored to other mechanisms of action combining other immuno-oncology (IO) therapies with either RT, or another IO therapy, or a targeted non-IO therapy, to explore corresponding anti-tumor efficacy in response to various dosing regimens and for varied baseline conditions of immuno-suppressive and immuno-activating markers of interest.

## Conclusions

The quantitative dynamic model described here characterizes the cancer immunity cycle and captures kinetic features of immune and tumor cell interactions in mouse CT26 tumors. More importantly, this model provides a basis for an in-silico evaluation tool to explore different RT and PD-L1 blockade combination regimens, suggesting that anti-PD-(L)1 treatment prior to, or concurrently with RT maximizes anti-tumor responses.

## Additional file

Additional file 1: Further information on model development and testing can be found in Additional file 1: the biological rationale for the proposed mathematical model structure; the structure of the mathematical model; population model development to describe inter-animal variability in tumor growth; model parameter estimations; model diagnostics; experimental data used for model development; model diagnostics; model validation against newly, independently generated sets of experimental tumor size data; design of efficacy simulations; a model sensitivity analysis. Additional file 1 also contains supplemental figures and references. (ZIP 6120 kb)

## Abbreviations

Ag: Antigen
APC: Antigen presenting cells
DAMP: Damage-associated molecular proteins
DC: Dendritic cells
IAR: Immune activation rate
IAV: Inter-animal variability
ICD: Immunogenic cell death
IFNɣ: Interferon-ɣ
IO: Immuno-oncology
ISC: Immuno-suppressive cells
mAbs: Monoclonal antibodies
NSCLC: Non-small cell lung cancer
PD-(L)1: Programmed death 1 (ligand)
QSP: Quantitative systems pharmacology
RT: Radiation therapy
TCD: Tumor cell death
TME: Tumor microenvironment
