# Supplementary material for: Radiation and PD-(L)1 treatment combinations: immune response and dose optimization via a predictive systems model
Source: J Immunother Cancer. 2018 Feb 27;6:17. doi: 10.1186/s40425-018-0327-9 (PMC5830328; doi:10.1186/s40425-018-0327-9)
Supplement: Supplementary file 1 — Further information on model development and testing can be found in Additional file 1: the biological rationale for the proposed mathematical model structure; the structure of the mathematical model; population model development to describe inter-animal variability in tumor growth; model parameter estimations; model diagnostics; experimental data used for model development; model diagnostics; model validation against newly, independently generated sets of experimental tumor size data; design of efficacy simulations; a model sensitivity analysis. Additional file 1 also contains supplemental figures and references. (ZIP 6120 kb) [file 40425_2018_327_MOESM1_ESM.zip › 2018-01-11-Paper-RT-IO-v17-Table_S2-Word.docx]

|  | | | | | | | | | | | | | | | | | | | | | | | | | | | |
| --- | --- | --- | --- | --- | --- | --- | --- | --- | --- | --- | --- | --- | --- | --- | --- | --- | --- | --- | --- | --- | --- | --- | --- | --- | --- | --- | --- |
| Efficacy, day 5 | | | | | | | Efficacy, day 7 | | | | | | | Efficacy, day 12 | | | | | | | Efficacy, day 19 | | | | | | |
|  | **No** | **aPD-L1, Day 3** | **aPD-L1, Day 5** | **aPD-L1, Day 7** | **aPD-L1, Day 12** | **aPD-L1, Day 19** |  | **No** | **aPD-L1, Day 3** | **aPD-L1, Day 5** | **aPD-L1, Day 7** | **aPD-L1, Day 12** | **aPD-L1, Day 19** |  | **No** | **aPD-L1, Day 3** | **aPD-L1, Day 5** | **aPD-L1, Day 7** | **aPD-L1, Day 12** | **aPD-L1, Day 19** |  | **No** | **aPD-L1, Day 3** | **aPD-L1, Day 5** | **aPD-L1, Day 7** | **aPD-L1, Day 12** | **aPD-L1, Day 19** |
| **No** | 0 (0-1) | 19 (13-25) | 17 (11-23) | 12 (7-18) | 3 (1-7) | 1 (0-2) | **No** | 0 (0-0) | 14 (8-20) | 13 (7-18) | 9 (5-14) | 2 (0-5) | 0 (0-1) | **No** | 0 (0-1) | 15 (10-21) | 14 (9-20) | 11 (6-16) | 3 (1-6) | 0 (0-2) | **No** | 0 (0-0) | 16 (11-23) | 14 (9-21) | 11 (5.5-16) | 2.5 (0-5) | 0 (0-2) |
| **RT 5Gy** | 10 (5-15) | 66 (58-74) | 66 (58-73) | 65 (57-72) | 48 (40-56) | 28 (21-35) | **RT 5Gy** | 5 (2-9) | 56 (47-64) | 55 (47-63) | 53 (45-62) | 39 (32-47) | 18 (11-24) | **RT 5Gy** | 2 (0-5) | 36 (28-44) | 34 (26-42) | 30 (23-38) | 18 (12-25) | 8 (4-12) | **RT 5Gy** | 0 (0-1) | 22 (16-29) | 20 (14-27) | 16 (11-23) | 6 (3-11) | 2 (0-5) |
| **RT 7Gy** | 27 (20-34) | 82 (76-88) | 82 (75-87) | 82 (75-88) | 79 (72-85) | 59 (50-67) | **RT 7Gy** | 17 (11-23) | 77 (70-84) | 76 (69-83) | 76 (68-83) | 69 (61-77) | 48 (40-57) | **RT 7Gy** | 5 (2-9) | 53 (44-61) | 52 (44-60) | 49 (41-57) | 37 (29-45) | 21.5 (15-29) | **RT 7Gy** | 1 (0-2) | 26 (19-33) | 25 (19-32) | 22 (15-29) | 10 (6-15) | 4 (1-7) |
| **RT 10Gy** | 36 (28-44) | 86 (80-92) | 86 (80-91.5) | 86 (80-91) | 84 (78-90) | 82 (76-88) | **RT 10Gy** | 31 (24-39) | 87 (81-92) | 86 (80-92) | 87 (81-92) | 86 (79-91) | 82 (76-89) | **RT 10Gy** | 21 (15-29) | 78 (71-85) | 77 (70-84) | 78 (72-85) | 75 (67-82) | 64 (56-71.5) | **RT 10Gy** | 2 (0-5) | 40 (32-48) | 38 (30-47) | 35 (28-43) | 23 (16-30) | 14 (9-20) |
| **RT 2x5Gy** | 31 (23-38) | 84 (78-90) | 84 (77-90) | 84 (78-90) | 83 (77-89) | 80 (73-86) | **RT 2x5Gy** | 26 (19-33) | 82 (76-88) | 82 (75-88) | 82 (75-88) | 80 (74-86) | 67 (59-74) | **RT 2x5Gy** | 9 (5-14) | 65 (56-73) | 64 (55-71) | 63 (55-70) | 52 (44-60) | 34 (27-41.5) | **RT 2x5Gy** | 1 (0-3) | 30 (23-38) | 29 (22-37) | 26 (19-34) | 13 (8-19.5) | 7 (3-11) |
| **RT 5x2Gy** | 25 (18-32) | 82 (75-88) | 81 (74-87) | 82 (75-88) | 76 (70-83) | 54 (46-63) | **RT 5x2Gy** | 13 (8-19.5) | 74 (66-81) | 73 (66-80) | 73 (65-79) | 63 (54-71) | 43 (35-51) | **RT 5x2Gy** | 4 (1-7) | 45 (37-54) | 44 (36-53) | 42 (34-50.5) | 28 (21-36) | 17 (11-23) | **RT 5x2Gy** | 0 (0-2) | 25 (18-32) | 23 (16-30) | 19 (13-26) | 8 (4-13) | 3 (0-6) |
|  | | | | | | | | | | | | | | | | | | | | | | | | | | | |
|  |  |  |  |  |  |  | DCm day 7 | | | | | | | DCm day 12 | | | | | | | DCm day 19 | | | | | | |
|  | **No** | **aPD-L1, Day 3** | **aPD-L1, Day 5** | **aPD-L1, Day 7** | **aPD-L1, Day 12** | **aPD-L1, Day 19** |  | **No** | **aPD-L1, Day 3** | **aPD-L1, Day 5** | **aPD-L1, Day 7** | **aPD-L1, Day 12** | **aPD-L1, Day 19** |  | **No** | **aPD-L1, Day 3** | **aPD-L1, Day 5** | **aPD-L1, Day 7** | **aPD-L1, Day 12** | **aPD-L1, Day 19** |  | **No** | **aPD-L1, Day 3** | **aPD-L1, Day 5** | **aPD-L1, Day 7** | **aPD-L1, Day 12** | **aPD-L1, Day 19** |
| **No** | 47 (29-65) | 58 (34-95) | 57 (34-95) | 56 (34-95) | 51 (33-75) | 47 (29-65) | **No** | 46 (30-63) | 57 (35-95) | 57 (35-95) | 56 (34-95) | 50 (33-71) | 46 (30-63) | **No** | 46 (30-65) | 56 (35-95) | 56 (35-95) | 55 (35-95) | 49 (34-74) | 46 (30-65) | **No** | 47 (28-65) | 58 (33-95) | 58 (33-95) | 57 (33-95) | 51 (32-74) | 47 (29-65) |
| **RT 5Gy** | 60 (47-85) | 83 (58-91) | 84 (58-91) | 85 (58-91) | 75 (54-92) | 61 (47-94) | **RT 5Gy** | 56 (41-78) | 79 (49-91) | 79 (49-91) | 79 (49-91) | 69 (47-92) | 56 (41-93) | **RT 5Gy** | 46 (36-67) | 60 (41-91) | 59 (41-90) | 58 (41-90) | 54 (40-89) | 48 (36-90) | **RT 5Gy** | 47 (33-65) | 58 (36-95) | 58 (36-95) | 57 (36-94) | 51 (35-84) | 47 (35-65) |
| **RT 7Gy** | 68 (53-83) | 80 (67-87) | 80 (66-87) | 81 (69-87) | 86 (64-89) | 90 (53-92) | **RT 7Gy** | 63 (47-83) | 76 (58-88) | 76 (57-88) | 76 (57-88) | 82 (55-90) | 73 (47-92) | **RT 7Gy** | 49 (39-80) | 71 (45-90) | 71 (45-89) | 70 (45-89) | 62 (44-89) | 56 (41-91) | **RT 7Gy** | 47 (37-65) | 58 (38-95) | 58 (38-95) | 57 (38-94) | 51 (38-85) | 47 (38-72) |
| **RT 10Gy** | 69 (51-77) | 63 (54-76) | 62 (54-75) | 63 (55-76) | 74 (51-79) | 84 (51-86) | **RT 10Gy** | 70 (56-79) | 65 (57-81) | 65 (56-80) | 62 (53-80) | 69 (61-82) | 81 (60-87) | **RT 10Gy** | 60 (45-82) | 70 (57-88) | 70 (57-87) | 70 (57-86) | 66 (55-86) | 71 (52-89) | **RT 10Gy** | 47 (40-68) | 62 (42-95) | 61 (42-95) | 59 (42-94) | 53 (41-85) | 50 (41-82) |
| **RT 2x5Gy** | 71 (56-81) | 73 (60-82) | 73 (60-82) | 74 (62-83) | 81 (63-86) | 88 (61-90) | **RT 2x5Gy** | 68 (50-82) | 72 (63-85) | 71 (63-85) | 71 (61-86) | 76 (62-87) | 86 (54-91) | **RT 2x5Gy** | 52 (41-81) | 71 (49-88) | 71 (49-88) | 70 (48-88) | 66 (47-88) | 62 (45-90) | **RT 2x5Gy** | 47 (38-65) | 58 (39-95) | 58 (39-95) | 57 (39-94) | 51 (39-84) | 47 (39-73) |
| **RT 5x2Gy** | 66 (50-83) | 75 (65-87) | 75 (65-87) | 76 (64-87) | 81 (61-89) | 87 (51-92) | **RT 5x2Gy** | 60 (44-83) | 73 (54-88) | 73 (54-88) | 73 (54-88) | 77 (52-90) | 70 (45-92) | **RT 5x2Gy** | 46 (38-75) | 66 (43-90) | 65 (42-89) | 63 (42-88) | 57 (42-88) | 52 (40-89) | **RT 5x2Gy** | 47 (34-65) | 58 (36-95) | 58 (36-95) | 57 (36-94) | 51 (36-84) | 47 (36-67) |
|  | | | | | | | | | | | | | | | | | | | | | | | | | | | |
| dTeff, day 5 | | | | | | | dTeff, day 7 | | | | | | | dTeff, day 12 | | | | | | | dTeff, day 19 | | | | | | |
|  | **No** | **aPD-L1, Day 3** | **aPD-L1, Day 5** | **aPD-L1, Day 7** | **aPD-L1, Day 12** | **aPD-L1, Day 19** |  | **No** | **aPD-L1, Day 3** | **aPD-L1, Day 5** | **aPD-L1, Day 7** | **aPD-L1, Day 12** | **aPD-L1, Day 19** |  | **No** | **aPD-L1, Day 3** | **aPD-L1, Day 5** | **aPD-L1, Day 7** | **aPD-L1, Day 12** | **aPD-L1, Day 19** |  | **No** | **aPD-L1, Day 3** | **aPD-L1, Day 5** | **aPD-L1, Day 7** | **aPD-L1, Day 12** | **aPD-L1, Day 19** |
| **No** | 178 (116-269) | 223 (131-1005) | 221 (131-1008) | 217 (131-1023) | 192 (128-340) | 178 (117-269) | **No** | 179 (120-263) | 225 (136-998) | 223 (136-999) | 219 (135-1014) | 193 (132-322) | 179 (120-263) | **No** | 178 (117-262) | 223 (133-998) | 222 (133-1006) | 217 (132-1014) | 192 (129-319) | 178 (117-262) | **No** | 182 (119-263) | 229 (135-1004) | 228 (135-1003) | 223 (134-1021) | 196 (131-322) | 182 (119-263) |
| **RT 5Gy** | 247 (177-541) | 765 (228-942) | 752 (228-955) | 758 (226-973) | 339 (208-1035) | 247 (177-1058) | **RT 5Gy** | 234 (156-528) | 764 (189-959) | 748 (189-969) | 750 (188-989) | 308 (180-1038) | 234 (156-1066) | **RT 5Gy** | 185 (126-297) | 272 (150-978) | 269 (150-981) | 261 (149-998) | 234 (145-1031) | 189 (132-1044) | **RT 5Gy** | 182 (119-263) | 229 (135-1007) | 228 (135-1012) | 223 (134-1024) | 196 (131-995) | 182 (119-263) |
| **RT 7Gy** | 303 (206-565) | 768 (294-868) | 761 (293-870) | 758 (292-912) | 781 (262-1015) | 767 (206-993) | **RT 7Gy** | 281 (182-561) | 807 (236-885) | 800 (236-892) | 810 (235-912) | 799 (224-1000) | 357 (182-1048) | **RT 7Gy** | 208 (139-508) | 692 (172-915) | 643 (171-915) | 362 (171-930) | 289 (167-972) | 240 (151-1059) | **RT 7Gy** | 182 (119-263) | 229 (135-1006) | 228 (135-1013) | 223 (134-1005) | 196 (131-970) | 182 (124-342) |
| **RT 10Gy** | 398 (247-504) | 673 (383-830) | 675 (382-831) | 688 (384-848) | 746 (391-882) | 644 (312-806) | **RT 10Gy** | 406 (238-529) | 729 (431-837) | 730 (427-837) | 743 (431-843) | 790 (392-889) | 809 (284-909) | **RT 10Gy** | 276 (175-567) | 787 (236-877) | 787 (235-864) | 800 (235-860) | 824 (228-864) | 815 (209-988) | **RT 10Gy** | 182 (128-303) | 297 (160-1006) | 289 (160-1013) | 276 (160-1005) | 236 (157-903) | 214 (153-963) |
| **RT 2x5Gy** | 355 (225-550) | 756 (363-852) | 758 (360-853) | 759 (360-869) | 786 (314-955) | 754 (242-934) | **RT 2x5Gy** | 323 (202-566) | 798 (280-846) | 798 (280-846) | 805 (279-855) | 816 (264-963) | 813 (208-1005) | **RT 2x5Gy** | 227 (149-562) | 809 (189-907) | 797 (189-883) | 795 (189-887) | 661 (183-923) | 285 (169-1034) | **RT 2x5Gy** | 182 (119-263) | 229 (137-1007) | 228 (136-1012) | 223 (137-1026) | 196 (135-939) | 182 (132-962) |
| **RT 5x2Gy** | 291 (197-568) | 792 (272-874) | 792 (271-882) | 798 (270-910) | 791 (250-997) | 758 (197-1028) | **RT 5x2Gy** | 264 (171-564) | 818 (220-894) | 815 (220-902) | 818 (219-919) | 803 (211-988) | 321 (171-1056) | **RT 5x2Gy** | 188 (127-343) | 322 (156-978) | 314 (156-965) | 300 (156-939) | 257 (152-968) | 224 (144-1049 | **RT 5x2Gy** | 182 (119-263) | 229 (135-1014) | 228 (135-1016) | 223 (134-1030) | 196 (131-957) | 182 (119-290) |
