# Supplementary material for: Radiation and PD-(L)1 treatment combinations: immune response and dose optimization via a predictive systems model
Source: J Immunother Cancer. 2018 Feb 27;6:17. doi: 10.1186/s40425-018-0327-9 (PMC5830328; doi:10.1186/s40425-018-0327-9)
Supplement: Supplementary file 1 — Further information on model development and testing can be found in Additional file 1: the biological rationale for the proposed mathematical model structure; the structure of the mathematical model; population model development to describe inter-animal variability in tumor growth; model parameter estimations; model diagnostics; experimental data used for model development; model diagnostics; model validation against newly, independently generated sets of experimental tumor size data; design of efficacy simulations; a model sensitivity analysis. Additional file 1 also contains supplemental figures and references. (ZIP 6120 kb) [file 40425_2018_327_MOESM1_ESM.zip › 2018-01-11-RT+IO Grouped SUPPLEMENTAL Figures_v17.pptx]

## Slide 1
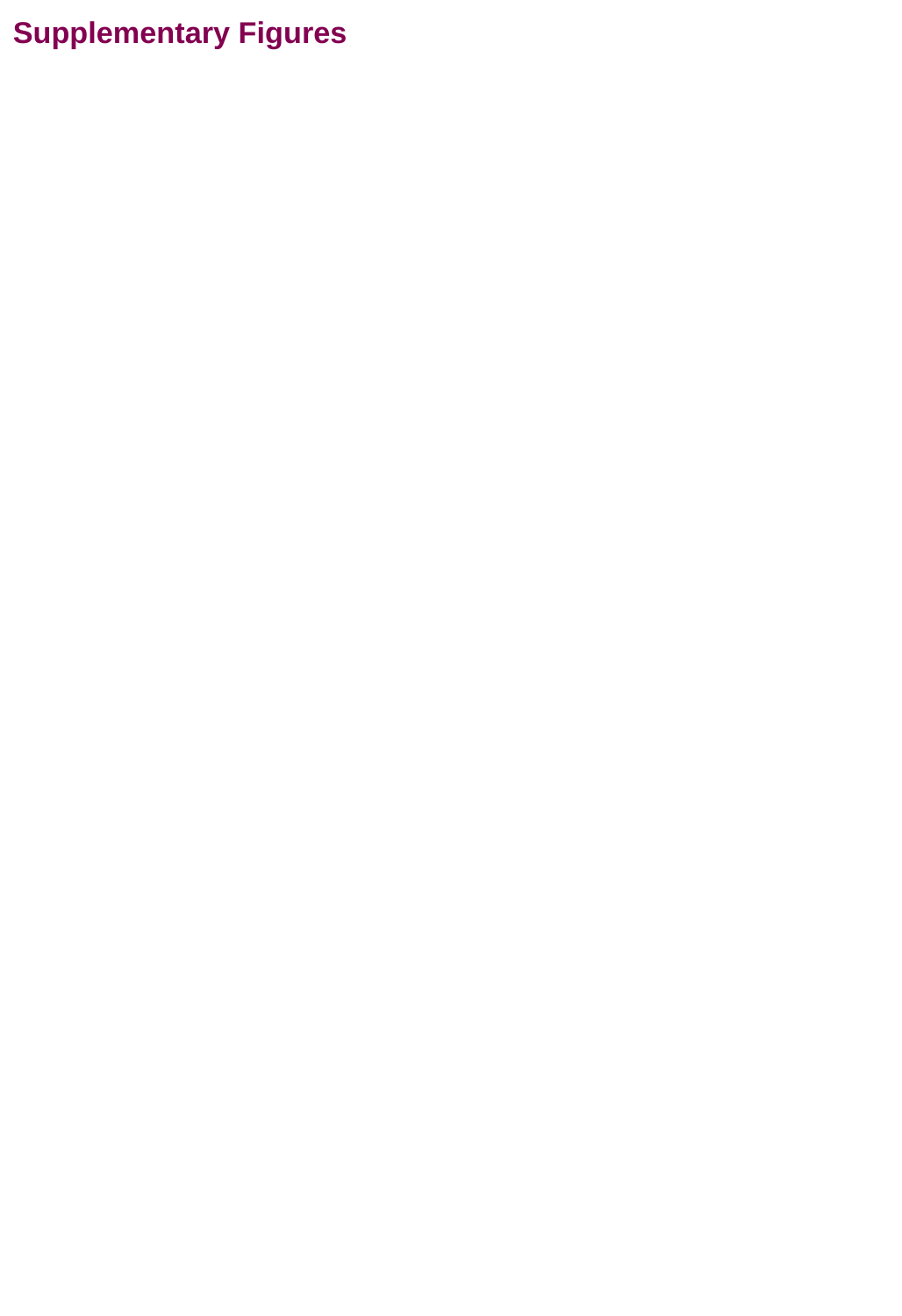

# Supplementary Figures
1

## Slide 2
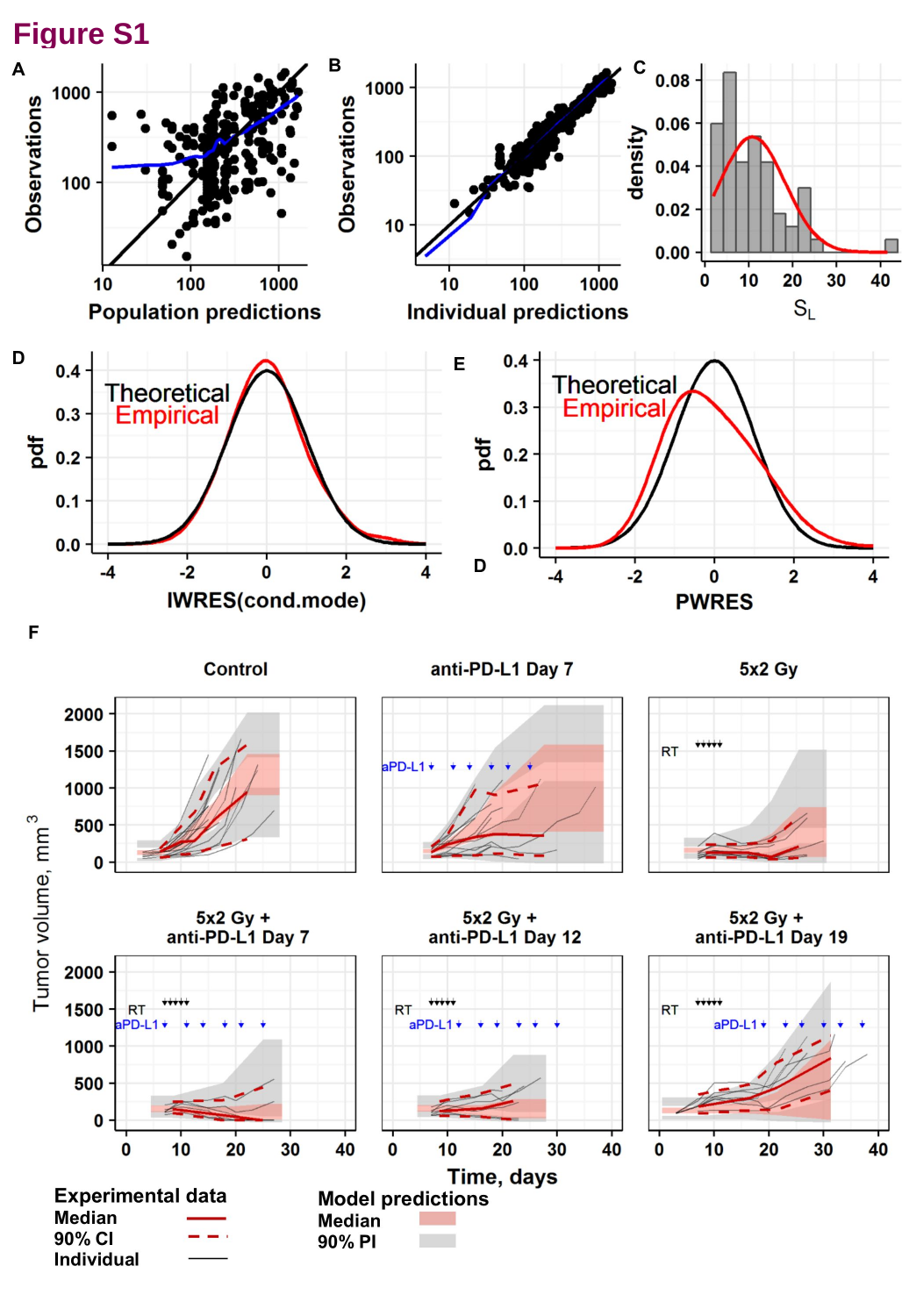

Figure S1

## Slide 3
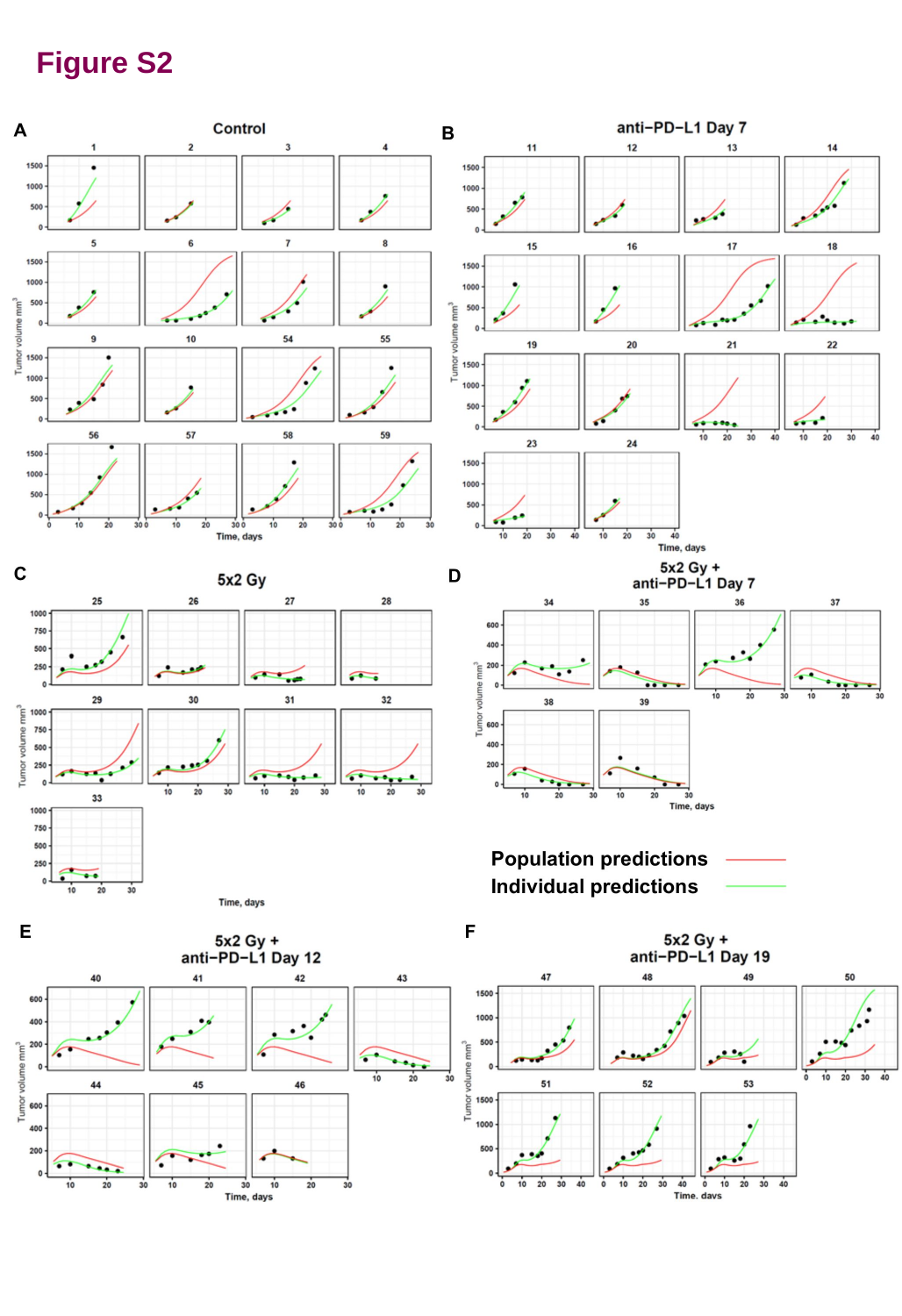

# Figure S2

## Slide 4
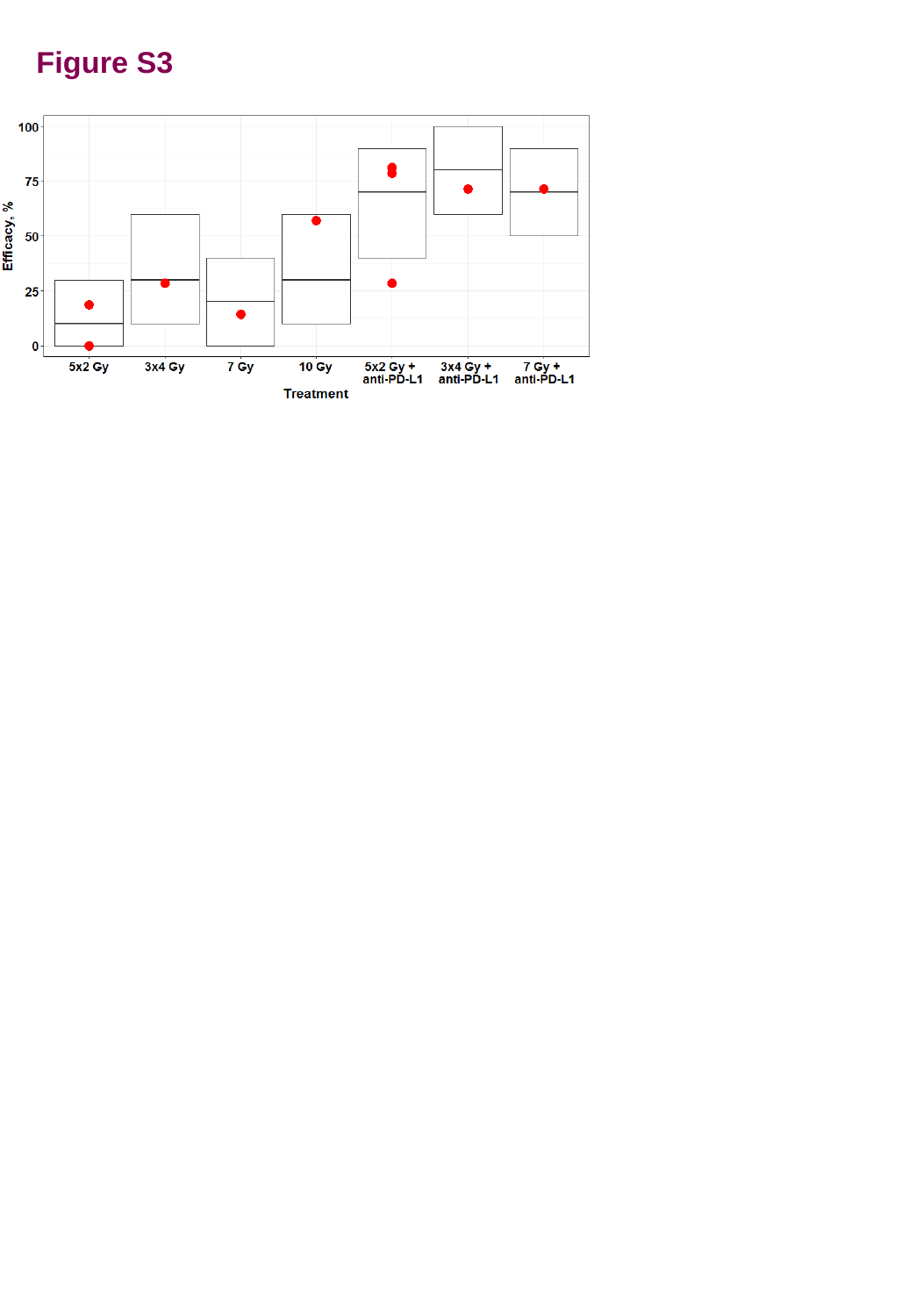

# Figure S3
4

## Slide 5
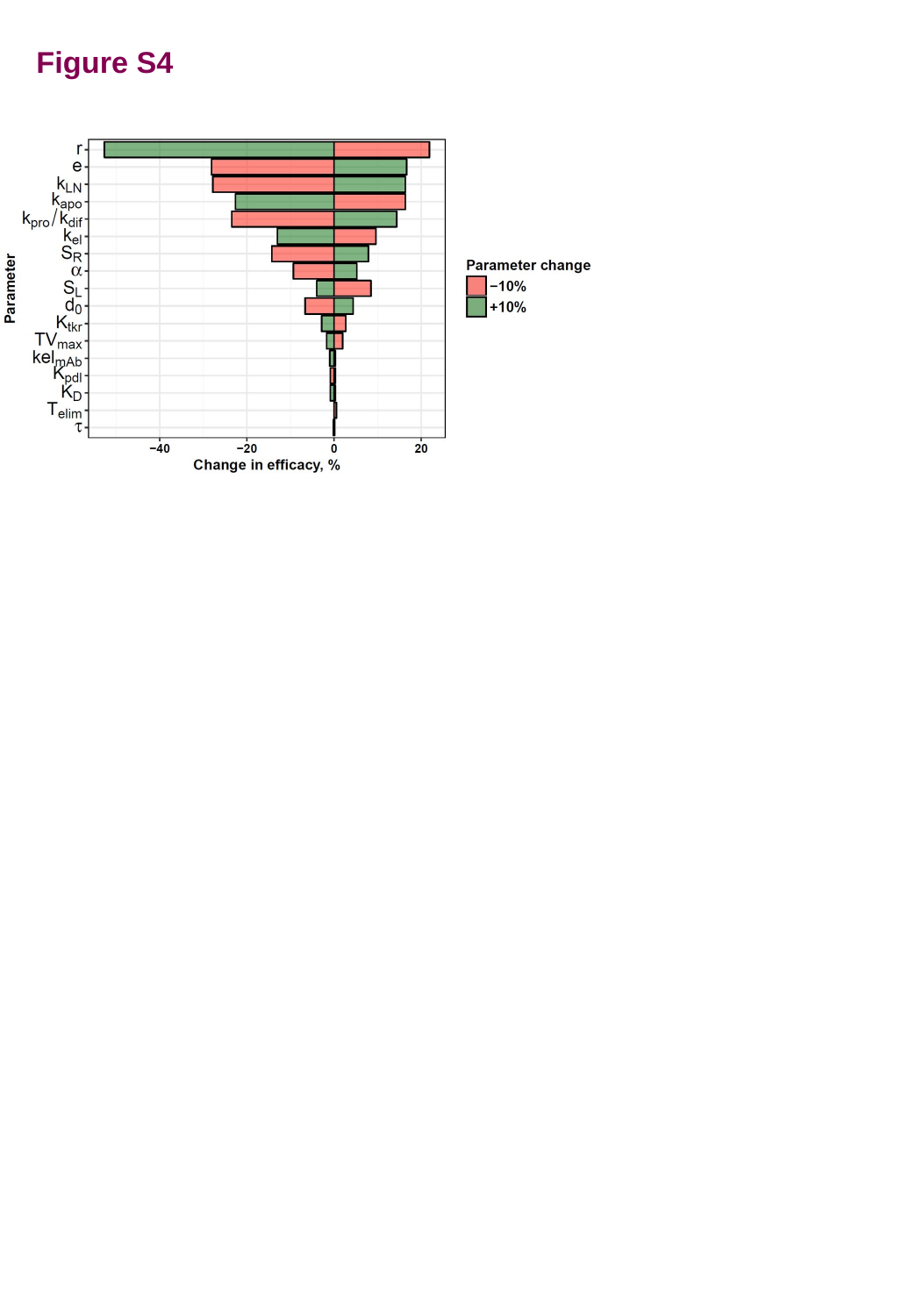

# Figure S4
5

## Slide 6
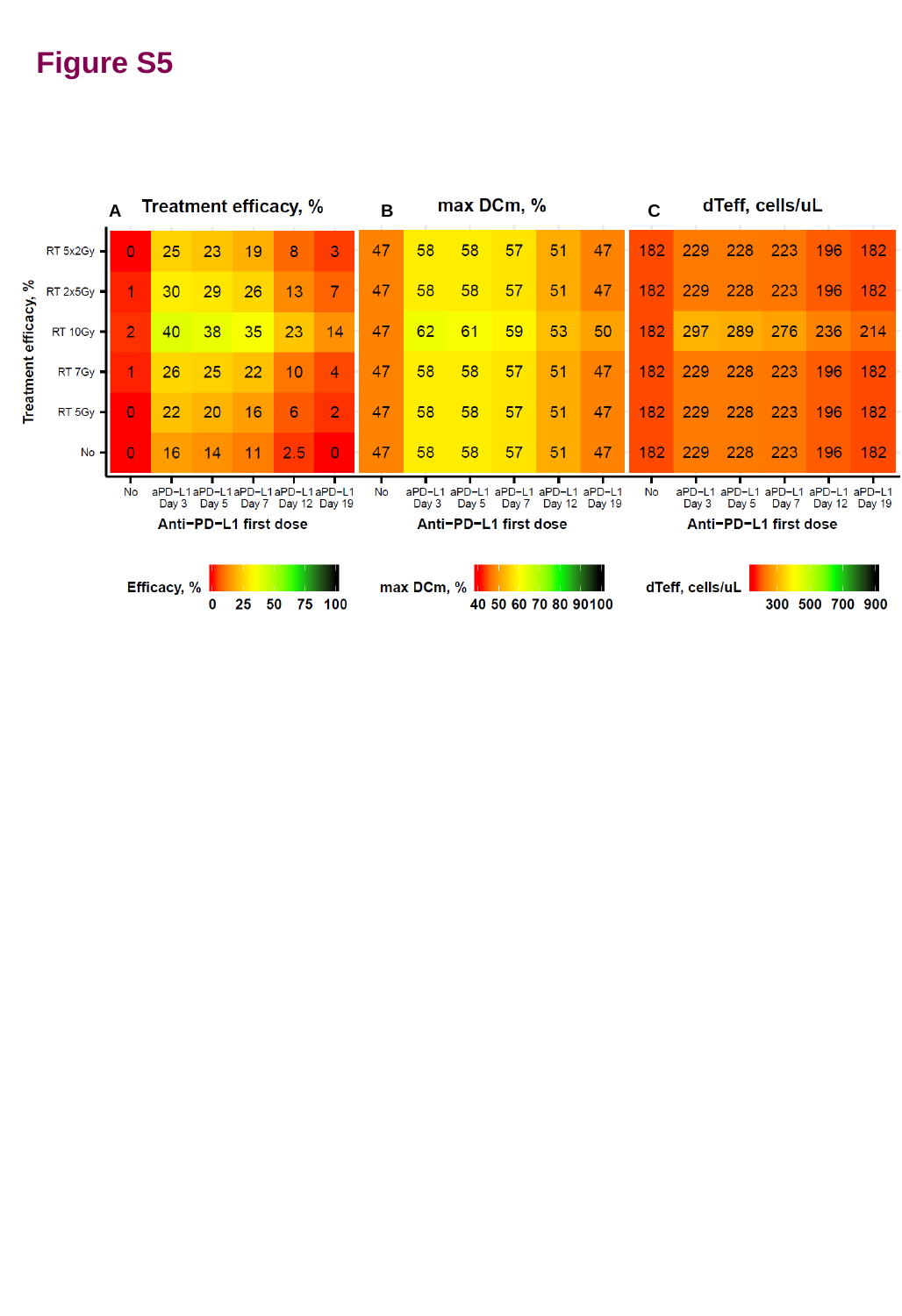

# Figure S5
A
B
C
6
