# Supplementary material for: Radiation and PD-(L)1 treatment combinations: immune response and dose optimization via a predictive systems model
Source: J Immunother Cancer. 2018 Feb 27;6:17. doi: 10.1186/s40425-018-0327-9 (PMC5830328; doi:10.1186/s40425-018-0327-9)
Supplement: Supplementary file 1 — Further information on model development and testing can be found in Additional file 1: the biological rationale for the proposed mathematical model structure; the structure of the mathematical model; population model development to describe inter-animal variability in tumor growth; model parameter estimations; model diagnostics; experimental data used for model development; model diagnostics; model validation against newly, independently generated sets of experimental tumor size data; design of efficacy simulations; a model sensitivity analysis. Additional file 1 also contains supplemental figures and references. (ZIP 6120 kb) [file 40425_2018_327_MOESM1_ESM.zip › 2018-02-09-RT+IO QSP SUPPLEMENTAL final_JITC.docx]

**Supplemental Information**

**Radiation and PD-(L)1 treatment combinations: Immune response and dose optimization *via* a predictive systems model**

Yuri Kosinsky**1**, Simon Dovedi**2**, Kirill Peskov**1**, Veronika Voronova**1**, Lulu Chu**3a**, Helen Tomkinson**3b**, Nidal Al-Huniti**3a**, Donald R Stanski**3c**, Gabriel Helmlinger**3a**

Correspondence: [gabriel.helmlinger@astrazeneca.com](mailto:gabriel.helmlinger@astrazeneca.com) (G.H.)

**S1. Biological rationale for the proposed mathematical model structure**

An exploratory evaluation of the experimental data, linked to a thorough literature-based understanding of biological mechanisms and assumptions were first necessary, in order to guide decisions on how to build and test the minimalistic, yet mechanistically-oriented quantitative systems pharmacology (QSP) model. The following key features emerged, and were considered later in the QSP model building process:

- 1. Individual tumor growth patterns in CT26
- In the control group, tumors grew exponentially without reaching a plateau during the study period. There is significant inter-animal variability (*IAV*) in tumor dynamics (growth patterns); furthermore, *IAV* differs across treatments [1].
- Anti PD-1 or anti PD-L1 monotherapies delayed tumor growth, yet only in a small sub-group of animals; such delays were also associated with anti-tumor immune responses. A majority of animals showed limited treatment benefits, with a significant number exhibiting exponential tumor growth, similarly to control conditions [1]
- Radiation (RT) monotherapy caused tumor shrinkage in all mice after Day 10. However, tumor re-growth started after Days 15 to 20, with no cases of complete tumor rejection [1].
- Combination treatments showed improved tumor shrinkage patterns in all dose sequences considered. The best response patterns were observed in the concurrent treatment setting, with two cases of complete tumor rejections [1].
  1. Immunological cell death and its effect on dendritic cell maturation
- Several studies suggested that the ability of RT-induced cell death triggering an immune response in the TME is a key factor in promoting the synergistic effects between RT and PD-(L)1 blockade [2,3]. Such an immune response in the TME results from effects downstream of *Immunogenic Cell Death* (*ICD*), which include a transient increase in tumor antigens following *ICD* and the subsequent release of DAMPs [4–6].
- RT-induced DAMPs release is dependent on radiation doses [7]. Extracellular DAMPs are detected by dendritic cells (DC), which enhance macrophage phagocytosis of tumor cells and antigen presentation [4–6]. For example, extracellular HMGB1 stimulates toll-like receptor 4 signaling in DC, enabling the DC maturation process and the ability of DC to prime tumor-antigen reactive CD8+ cells [8–11].
  1. Time course of PD-L1 expression levels
  - PD-L1 expression levels measured by median fluorescence intensity (MFI) present a transient increase in response to RT treatment, as shown by Dovedi et al [12], suggesting that an immune response is activated transiently in the TME, with a duration of approximately 3 days following completion of the RT treatment cycle (or on Day 14 following tumor cell inoculation).
  - One of the key drivers for PD-L1 expression is the release of interferon-y (IFNy) by activated immune cells. It has indeed been shown that, *in vitro*, IFNy induces tumor cell PD-L1 expression in a dose-dependent manner, with a characteristic time of about 24 hours [13]. This IFNy to PD-L1 relationship was also demonstrated *in vivo* in CT26 tumors [12].
  - Several preclinical studies have shown that PD-1/PD-L1 interactions suppress T cell proliferation, pro-inflammatory cytokine production, and antigen-dependent cytotoxicity [14]. Therefore, in the model, immuno-suppression should be represented as a negative feedback mechanism, driven by immune response activation and an increase in PD-L1 expression.
  1. *CD8+:Treg* ratio: Time-dependent changes
  - Another important immuno-suppression mechanism relates to the slow accumulation of regulatory T cells (*Treg*), myeloid-derived suppressor cells (MDSC), and other immuno-suppressive cell types, which mediates tumor re-growth, 18 to 20 days following tumor cell inoculation. In preclinical experiments and under control conditions, the ratio of *CD8+:Treg* cell counts decreases over time, suggesting an elevation of Treg in the TME over time [15].
  - Furthermore, CD4+ cell depletion (both Th1/2 and Treg) significantly increases PD-L1 expression by RT and enhances anti-tumor responses, whereas CD8+ cell depletion completely abrogates RT effects on PD-L1 upregulation - as measured *via* mean fluorescence intensity, *MFI* [1].
  - Additionally, syngeneic CT26 tumors, as compared to other experimental murine models, are characterized by a higher baseline of Treg cell count in tumor tissue [16], showing the importance of this immuno-suppressive mechanism in CT26. Analogous findings were also shown in other syngeneic models [17]. Based on these collective preclinical data, the accumulation of Treg in the TME and the subsequent decrease in the *CD8+:Treg* ratio were determined to be additional key immuno-suppressive mechanisms critical for sustaining tumor growth.

The above key features were selected to determine the key physical molecular and cellular players as well as the quantitative functions of immuno-activation and immuno-suppression of the cancer immunity cycle, to be represented in the QSP model.

**S2. Structure of the mathematical model**

***Intrinsic tumor growth model***

The intrinsic growth dynamics (a volume, in μL) of CT26 tumors were well-described by a logistic equation, according to Equation 1:

dTV/dt = r • TV • (1– TV/TVmax) (1)

where *TV* is the tumor volume and *r, TVmax*are parameters describing the intrinsic tumor growth rate and the upper limit of tumor size, respectively.

***Direct effect of RT on tumor cells***

As already mentioned, RT treatment was assumed to affect highly proliferating tumor cells only, given the relatively low RT doses used in the experiments (equal or below a single 10 Gy dose of radiation). A linear-quadratic (LQ) approach is most commonly used in radiobiology [18], to describe cellular responses to radiation. This LQ equation (Equation 2) stems from a mechanistically-based model for cell kill and sublethal damage repair and is expressed, for a single acute radiation dose, as:

ln(N/N0) = - α • D – β • D2 (2)

where *D* (in Gy) is the radiation dose, *N0* is the number of cells initially present, *N* is the number of surviving cells, and *α, β* are coefficients describing the linear and quadratic portions of the survival curve. However, this equation does not take into account any cell proliferation process. We thus adopted ordinary differential equations (ODEs) from [19], which build upon the LQ equation with minor updates (Equations 3 to 5). An intermediate compartment *TVd* representing the volume of RT-damaged pre-apoptotic cells is thus added, to account for their contribution towards the observed tumor volume, as described in [20].

dU/dt = δ • R(t) – U/τ (3)

dTV/dt = r • TV • (1– TV/TVmax) - (α • R(t) + κ • U2) • TV (4)

dTVd/dt = (α • R(t) + κ • U2) • TV - μ • TVd (5)

Here, *R(t)* describes the radiation dose rate within a finite time interval; it allows for the model to describe any RT dosing regimen. In our experiments, relatively small RT doses were applied locally to the tumor (5 x 2 Gy dose, 7 Gy single dose). *U(t)* represents the average number of double-strand breaks (DSBs) per cell.

The observed tumor volume is:

TVtot = TV + TVd (6)

As shown in [19], integrating the ODEs yields the LQ equation with the following expression for parameter *β*:

β = δ2 • κ • τ /2 (7)

A high ratio of *α/β = 10 (1/Gy)* is typical for tumor cells, as discussed in [21]. Parameters in Equation 7 can be constrained or estimated using available literature data (see Table 1). Equation 7 may be rearranged to yield *κ*:

κ = 2•(α/10) / δ2 • τ (8)

The remaining parameter *α 1/Gy *can then be fitted using CT26 tumor growth dynamics data. A half-life estimate of 4 days was used for RT-damaged tumor cells [20], to then estimate the elimination rate of RT damaged tumor cells, *μ (d-1)*.

***Modeling of T cells within the cancer immunity cycle***

In the QSP model, immune cells were assumed to distribute homogeneously throughout the tumor. The first step of the cancer immunity cycle involves tumor cell death (*TCD*) (Step 1, Figure 1A). In order to take immune-dependent effects of tumor inhibition into the description of tumor size dynamics, the rate of change of tumor volume (Equation 4) was modified to yield Equation 9:

dTV/dt = r•TV•(1– TV/TVmax) - (α•R + •U2)•TV – (e•dTeff + d 0)•TV (9)

where *e•dTeff* represents the tumor cell elimination rate, governed by *dTeff*, with *e* as an efficacy coefficient. *d0*is a slow constant rate representing other processes leading to tumor cell kill, *e.g*., *via* natural killer cells. Overall, a *TCD* rate function (*d-1*), which summarizes different mechanisms leading to tumor cell death, was characterized by Equation 10:

TCD =  • TVd / TVtot + e • dTeff + d 0 (10)

Next, an immune response by *ICD*, *e.g*., *via* various DAMPs, builds up in response to tumor cell death, leading to DC maturation (Step 2, Figure 1A) and stimulation of the antigen-presenting process (Step 3, Figure 1A).

DC maturation was captured in the model using a variable *DCm*, expressed as an Emax function of *TCD*, as shown in Equation 11:

DCm = TCD2 / (TCD2 + Ktcd2) (11)

where *Ktcd* is the sensitivity of DC maturation to local *TCD*.

The stimulation of *Agsys*, the level of tumor antigen presentation, quantitatively depends on both the level of DC maturation and the intrinsic tumor volume, as described by Equation 12:

Agsys = DCm • TVtot (12)

Two forms of T cells were taken into account in the model, according to the current view of T cell development and as reviewed in [22,23]: (1) non-differentiated *nTeff*-relatively long-lived T cells, which may proliferate and differentiate to (2) terminally differentiated, cytotoxic effector T cells, *dTeff* (Step 5, Figure 1A). *dTeff* are shorter-lived and, while losing proliferative potential, gain effector and homing competencies.

Elevation in *Agsys* drives the influx of primed, non-differentiated T cells (*nTeff*) from the systemic circulation into tumor tissue, per unit of tumor volume (Step 4, Figure 1A); antigen-driven *nTeff* influx was assumed to follow a Michaelis-Menten form and expressed as a function of the systemic level of antigen (Equation 13). T cell proliferation and differentiation take place within tumor tissue and are regulated by properties of the TME. The time rate of change for *nTeff* and *dTeff* are described by Equations 13 and 14:

dnTeff /dt = kLN • Agsys/(Agsys + SL) + IAR • nTeff •(kpro - kdif) - kel • nTeff (13)

ddTeff/dt = IAR • nTeff • kdif - kapo • dTeff (14)

In Equation 13, *kLN* represents the maximal influx rate of *nTeff* cells, while *SL* is the sensitivity of non-differentiated T cell influx that is driven by *Agsys*.*kpro*and *kdif* are rate constants of, respectively, T cell proliferation and differentiation. *kel*and *kapo*are, respectively, the slow and fast elimination rates for *nTeff*and *dTeff* [24]. *kdif*was then constrained to prevent unlimited growth of *nTeff*within the tumor, thereby satisfying a relationship of *kdif > (kpro - kel)*. Thus, the *dTeff* cell count in tumor depends on both the influx of *nTeff* from the systemic circulation and the T cell activation process through proliferation and differentiation within tumor tissue. Activated cytotoxic *dTeff* cells subsequently eliminate tumor cells through an increase in *TCD*, which allows for further influx of *nTeff*.

The T cell activation process is dependent on an *Immune Activation Rate* function (*IAR*, in Equations 13 to 17), which may take values between 0 and 1 and integrates the effects of two important immuno-suppression components in the model, namely PD-L1 expression (*PD-L1*) and immune suppressive cells accumulation (*ISC*), as shown in Equation 15.

IAR = (1- PD-L1) • (1 – ISC) (15)

If *IAR* is high (*e.g*., close to a value of *1*), *dTeff* become the dominant form of T cells in the TME, leading to effective anti-tumor response. If *IAR* is, instead, close to *0*, *nTeff* may accumulate in the TME, which would result in a limited anti-tumor response.

One key immuno-suppressive mechanism is driven by the overall level of PD-L1 expression in tumor tissue. Effector T cells produce IFN-γ, a well-known inducer of PD-L1 expression on tumor cells and tumor-infiltrated myeloid cells [25]. Given limitations in experimental data and in order to preserve model identifiability, we simplified and defined the variable *PD-L1* to represent the PD-L1-mediated suppressive effect on T cells (Equations 16; Step 8, Section 3.1). Therefore, the *PD-L1* model variable is not directly proportional to the physical concentration of PD-L1 concentration in the TME.

dPD-L1/dt = kPDL • (dTeff / (dTeff + KPDL) – PD-L1) (16)

*KPDL* is a sensitivity parameter of *PDL1* induced by *dTeff*; *kPDL* is the rate of PD-L1 response development, which was assumed to reach a steady state in about 1 day, as shown *in vitro* [13].

In order to incorporate the effects of anti PD-L1/anti PD-1 antibody (mAb) in the immune cancer cycle, a pharmacokinetics (PK) model with first-order absorption and linear elimination was used to describe the plasma pharmacokinetics of the *i.p.* administered PD-L1 mAb [26], and used as a target-modulating driving function in the QSP model. The level of *PD-L1free* was expressed as shown in Equation 17:

PD-L1free = PD-L1 / (1 + mAb/KD) (17)

*mAb* is the drug concentration in plasma and *KD (nM)* represents the antibody dissociation constant.

To take into account anti PD-L1 antibody effects, Equation 15 describing *IAR*was modified to yield Equation 18:

IAR = (1- PD-L1free) • (1 – ISC) (18)

In the current model, we do not specify any differences in the mechanisms of action between PD-1 and PD-L1 blocking antibodies; therefore both types of antibodies directly decrease *PD-L1free* (Equation 17). To our knowledge, no notable differences in clinical efficacy were observed between anti PD-1 or anti PD-L1 treatments, despite different downstream signaling pathways following each type of blockade [27].

In addition, and in order to compare simulated PD-L1 against experimentally measured PD-L1 values, we introduce *PD-L1MFI* in the model, which corresponds to measured mean fluorescence intensities of *PD-L1* on tumor cells, using Equation 19:

PD-L1MFI = PD-L1 / (1 - PD-L1) (19)

Another key immuno-suppressive mechanism that governs *IAR* is the level of accumulated *ISC* in tumor tissue. Increased *Agsys* also drives the*ISC* accumulation process, which may be characterized by Equation 20:

ISC = Agsys / (Agsys + sR) (20)

where *SR* is a parameter responsible for the sensitivity of immune suppressive cells tumor infiltration in response to systemic antigen exposure. It should be noted that modulation of TME immuno-suppressive properties is a complex process that involves different cell types and cytokines, *e.g.*, MDSC or type 2 tumor-associated tumor macrophages (TAM). Since we had limited information on the detailed accumulation kinetics of these various cell types during CT26 tumor growth *in vivo* [28], we considered *ISC* as a driving function in the model; it may be interpreted as a lumped representation summarizing these multiple mechanisms of TME immuno-suppressive conditions.

Overall, our model successfully captured key features of the cancer immunity cycle [25], in minimalistic yet mechanistic terms.

***Population model development to describe inter-animal variability in tumor growth***

As discussed earlier, high *IAV* in tumor growth was experimentally observed in each of the treatment groups. Preclinical data strongly supported the hypothesis that activated CD8+ T cell density in the TME plays an important role in determining tumor growth or shrinkage rates[1,17,29–34]. Therefore, the ability of T cells to infiltrate tumor tissue under systemic antigen exposure, *SL*, was determined to drive *IAV* and assumed to follow a log-normal distribution:

SL(i) = SL • exp(ηi) (21)

where *ηi*is a random effect value normally distributed with a mean of *0* and a standard deviation of *Ω_SL*.

Other biological mechanisms that potentially underlie *IAV* were tested as well; results are shown in Table S2. A random effect was applied to different model parameters related to the T cell turnover function (*SL, e, r*), yet the best quality-of-fit for *IAV* was obtained when applying the random effect on *SL*.

Also, several residual error models were tested for *TVtot* model, and the combined residual error model was chosen as follows, based on tumor size data fitting:

TVtotobs i,j = TVtotpred i,j + (a + b • TVtotpred i,j ) • ɛi,j (22)

where *TVtotobs i,j* and *TVtotpred i,j* represent the observed and predicted *TVtot* values, respectively, for animal *i* at time *j*. *ɛ****i,j*** is an independent random variable normally distributed with a mean of *0* and a standard deviation of *1*.

**S3. Model parameter estimations**

A total of 25 parameters were used (Table 1), of which 13 were estimated using values from the literature. 4 of the remaining parameters were fixed based on reasonable biological ranges. Values for the remaining 8 parameters (5 population means + 1 random effect + 2 residual error parameters) were fitted using a maximum likelihood method. In order to achieve the best model fitting to individual tumor size dynamics, model quality was evaluated using multiple criteria: (i) change in the objective function value (OFV) (logarithm of likelihood, Akaike information criterion), (ii) inspection of diagnostic plots, (iii) precision and identifiability of parameter estimates (based on estimated relative standard error values, or RSE: standard error divided by the mean and expressed as a percentage), as well as (iv) minimization of *IAV* and the residual error (*a,b*).

An initial set of parameters for model calibration was chosen based on physiological limits available from literature sources. Based on these limits, 5 sets of physiologically-plausible initial values were randomly generated, and parameter estimation was performed for each set of initial values. Estimated parameter values did not depend significantly on initial values, which also supported model identifiability.

**S4. Model diagnostics**

The model was calibrated based on CT26 tumor size dynamics in individual animals, under RT and anti PD-L1 treatments (monotherapies and combination treatments: RT + anti PD-L1 on Day 7; RT+ anti PD-L1 on Day 12; RT + anti PD-L1 on Day 19), using a nonlinear mixed-effects modeling procedure. Model fits for individual tumor size dynamics and average trends under all treatment conditions were examined. Figure 1B shows model-based median (red solid lines) with 90% prediction intervals (red-shaded areas) for all experimental groups. Experimental tumor size data were then mapped onto predictions; most of the data were captured within the 10-90% percentile interval (grey lines, Figure 1B). In addition, a model diagnostic plots showed that the average population and individual trends of tumor dynamics were adequately described by the model (Figures S1, S2).

**S5. Model validation against newly, independently generated sets of experimental tumor size data**

The predictive power of the QSP model was assessed *via* an external cross validation: the model was used in a forward-simulation mode, by simulating new experimental scenarios for which tumor size data had been independently generated, to indeed determine whether we could predict such data – data which had not been used in the model development and evaluation steps described above. The following scenarios were simulated for this purpose, with a post-hoc verification against the existing data [1,12]: (1) single 7 Gy dose RT alone and in combination with anti-PD-L1 (10 mg/kg 3qw); (2) fractionated 3x4 Gy RT alone and in combination with anti-PD-L1 (10 mg/kg 3qw); (3) combination therapy of fractionated 5x2 Gy RT and anti-PD-L1 treatment (10 mg/kg 3qw), with and without administration of an anti-CD8 antibody; (4) PD-L1 expression levels upon RT alone (5x2 Gy) and RT + anti-PD-1 combination.

The QSP model adequately reproduced all these additional experimental data (Figure 2), demonstrating its ability to predict individual tumor responses to *de novo* mono- and combination treatment regimens, as well as to provide mechanistic insights of the underlying molecular and cellular dynamical interplays in tumor tissue such as *dTeff*, immuno-suppressive forces, levels of PD-L1 expression.

Figure 2A shows that the model adequately predicted CT26 tumor size dynamics, for either single dose (7 Gy) or fractionated (3x4Gy) RT administered alone or in combination with an anti-PD-L1 antibody (10 mg/kg 3qw). The predicted distributions of tumor sizes for either RT alone or combination treatment, along with the corresponding predicted median values were consistent with experimental measurements; most of the individual tumor size data fell within the model 90% prediction interval (Figure 2A).

The QSP model was next used to predict the effects of CD8+ *dTeff* depletion on tumor size response, upon administration of an anti-CD8 mAb. Previous studies in multiple murine experiments involving RT + IO combination treatments have demonstrated that, upon CD8+ T cell depletion, RT-dependent anti-tumor immune responses are dramatically reduced [1,17,29,31–36]. This anti-CD8 mAb effect was simulated in the QSP model by decreasing parameter *kLN*, the maximal T-cell infiltration rate, by a factor of 10 (specifically, on Day 7 in the combination setting of fractionated 5x2 Gy RT + anti-PD-L1 mAb). The model demonstrates that CD8+ T-cell depletion led to faster tumor growth, highly comparable to the control setting (Figure 2B). As mentioned previously, the combination treatment without the anti-CD8 mAb produced a pronounced anti-tumor immune response, greatly delaying tumor growth (down to about 100 µL by Day 13) *vs*. control. In the presence of an anti-CD8 mAb, anti-tumor responses were nearly eliminated (Figure 2B), thereby matching experimental measurements [1]. In the context of an established solid tumor setting, these data further support the hypothesis that T-cell influx and *dTeff* activity within tumor tissue play a critical role in modulating anti-tumor effects of RT [17,32,34].

PD-L1 expression levels on tumor cells have been suggested to reflect local TME changes and are measured in preclinical studies, typically to investigate negative feedback mechanisms which may contribute to tumor resistance [37]. Therefore, capturing the correct PD-L1 expression levels following RT is important in characterizing this immuno-suppressive mechanism. For comparison with model predictions, all PD-L1 measurements [1] were normalized against the control group measurements. Model predictions of PD-L1 expression levels were based on simulations of control, RT mono- and RT + anti-PD-1 combination treatments on Day 12 to 18 and were in agreement with experimental measurements (Figure 2C): higher levels of PD-L1 expression were predicted under RT mono- and concurrent RT + anti-PD-1 combination therapy, reflecting further enhancement of the T cell response. High IAV of PD-L1 expression levels were predicted for both mono- and combination therapies, consistent with the high variability in *dTeff* activation observed in tumor tissue and upon treatment.

Taken together, our external cross-validation based on new scenarios and verified against independently generated experimental data demonstrate the predictive power of the QSP model, which may thus be used to simulate the outcome of untested RT and RT + anti-PD-L1 combination treatment regimens. Such a mechanistically-based model, which captures the role of tumor tissue, CD8+ cells among other features, may be further used to explore the dynamical interplay of the underlying cellular and molecular biology *in vivo*, in response to RT + IO agents in syngeneic mouse experiments.

**S6. Design of Efficacy Simulations**

One way to evaluate efficacy comprehensively is to look at individual animal responses, rather than only cohort medians or means, and define a percentage of ‘responders’ based on animals showing complete tumor response after a given form of treatment. To test the QSP model in this respect, and in order to use an efficacy criterion comparable across treatments, we first defined a ‘responder’ as an individual animal with a tumor volume of ≤10 mm3 at Day 50 of treatment. Efficacy of a treatment cohort was then expressed as the percentage of responders, and could be compared to the same efficacy criterion in other simulated treatments.

We explored anti-tumor efficacy for two fractionated (5x2 Gy and 3x4 Gy) and two single dose (7 Gy and 10 Gy) RT monotherapies, and in combination with anti PD-L1 10 mg/kg 3qw (Figure S3). *Per* treatment scenario, we simulated a total of 2000 virtual experiments, with each experiment to include 10 animals, to match the typical size of actual experimental groups while taking into account both uncertainty and variability around of model parameters (Table 1). The median efficacy for each treatment scenario was estimated with a 90% confidence interval (CI), for comparisons against actual experimental efficacy data. Monotherapies of fractionated 5x2 Gy *vs*. single-dose 7 Gy RT demonstrated similar and relatively low efficacy (~10-20% responders), in agreement with experimental measurements from [12]The model predicted higher efficacy (~30% responders) for fractionated 3x4 Gy RT or single-dose 10 Gy RT. Experimental efficacy for single-dose 10 Gy RT was actually higher (~60% responders), but still within the 90% CI range of our model predictions. All three combination therapies of RT (either fractionated or single dose) with an anti PD-L1 mAb greatly improved median efficacy (up to ~80% responders), by a factor of 2 to 6 *vs*. monotherapies, again consistent with experimental data. No significant efficacy differences were observed between “biologically equivalent” fractionated 5x2 Gy RT *vs*. single-dose 7Gy RT, whether in mono- or combination settings.

Prospective simulation studies presented in this work were hence crucial to further understand the key biological processes underlying potential synergistic effects of RT + anti PD-(L)1 combination therapies, in order to optimize dose regimens for these treatment modalities. Overall, model predictions using the proposed efficacy criterion were robust and consistent across available experimental data [12], demonstrating the high predictive power of this QSP model and its potential use for prospective simulations of untested monotherapies or combination treatment scenarios.

**S7. Model sensitivity analysis**

In order to better understand the role of each model parameter and its potential impact on efficacy (as defined above), a parameter sensitivity analysis was conducted, by increasing or decreasing each parameter value, individually, by +/- 10% around a median value. Only values for *kpro* and *kdif* were varied simultaneously, since these parameters were strongly coupled given the model structure. Figure S4 shows that model predictions of efficacy (as defined above; % responders) were highly sensitive to the intrinsic tumor growth rate *r* and to parameters which relate to tumor tissue infiltration by T cells (*kLN, sL*), cytotoxic activity (*e*), and turnover processes (*kpro/kdif, kapo*). Other parameters provided only a moderate modulation of overall efficacy, such as *do* and *Ktcd* (which were given fixed values in the model); the remaining fixed parameters proved to be nearly insensitive towards overall efficacy.

**Supplemental References**

1. Dovedi SJ, Adlard AL, Lipowska-Bhalla G, McKenna C, Jones S, Cheadle EJ, et al. Acquired Resistance to Fractionated Radiotherapy Can Be Overcome by Concurrent PD-L1 Blockade. Cancer Res. 2014;74:5458–68.

2. Reits EA, Hodge JW, Herberts CA, Groothuis TA, Chakraborty M, K.Wansley E, et al. Radiation modulates the peptide repertoire, enhances MHC class I expression, and induces successful antitumor immunotherapy. J. Exp. Med. 2006;203:1259–71.

3. Zhang B, Bowerman NA, Salama JK, Schmidt H, Spiotto MT, Schietinger A, et al. Induced sensitization of tumor stroma leads to eradication of established cancer by T cells. J. Exp. Med. 2007;204:49–55.

4. Demaria S, Golden EB, Formenti SC. Role of Local Radiation Therapy in Cancer Immunotherapy. JAMA Oncol. 2015;1:1325.

5. Formenti SC, Demaria S. Systemic effects of local radiotherapy. Lancet Oncol. 2009;10:718–26.

6. Golden EB, Apetoh L. Radiotherapy and Immunogenic Cell Death. Semin. Radiat. Oncol. 2015;25:11–7.

7. Golden EB, Frances D, Pellicciotta I, Demaria S, Helen Barcellos-Hoff M, Formenti SC. Radiation fosters dose-dependent and chemotherapy-induced immunogenic cell death. OncoImmunology. 2014;3:e28518.

8. Aguilera TA, Rafat M, Castellini L, Shehade H, Kariolis MS, Hui AB-Y, et al. Reprogramming the immunological microenvironment through radiation and targeting Axl. Nat. Commun. 2016;7:13898.

9. Apetoh L, Ghiringhelli F, Tesniere A, Obeid M, Ortiz C, Criollo A, et al. Toll-like receptor 4–dependent contribution of the immune system to anticancer chemotherapy and radiotherapy. Nat. Med. 2007;13:1050–9.

10. Gupta A, Probst HC, Vuong V, Landshammer A, Muth S, Yagita H, et al. Radiotherapy Promotes Tumor-Specific Effector CD8+ T Cells via Dendritic Cell Activation. J. Immunol. 2012;189:558–66.

11. Schaue D, Ratikan JA, Iwamoto KS, McBride WH. Maximizing Tumor Immunity With Fractionated Radiation. Int. J. Radiat. Oncol. 2012;83:1306–10.

12. Dovedi SJ, Cheadle EJ, Popple A, Poon E, Morrow M, Stewart R, et al. Fractionated radiation therapy stimulates anti-tumor immunity mediated by both resident and infiltrating polyclonal T-cell populations when combined with PD1 blockade. Clin. Cancer Res. 2017;clincanres.1673.2016.

13. Gowrishankar K, Gunatilake D, Gallagher SJ, Tiffen J, Rizos H, Hersey P. Inducible but Not Constitutive Expression of PD-L1 in Human Melanoma Cells Is Dependent on Activation of NF-κB. Cheriyath V, editor. PLOS ONE. 2015;10:e0123410.

14. Wang C, Thudium KB, Han M, Wang X-T, Huang H, Feingersh D, et al. In Vitro Characterization of the Anti-PD-1 Antibody Nivolumab, BMS-936558, and In Vivo Toxicology in Non-Human Primates. Cancer Immunol. Res. 2014;2:846–56.

15. Twyman-Saint Victor C, Rech AJ, Maity A, Rengan R, Pauken KE, Stelekati E, et al. Radiation and dual checkpoint blockade activate non-redundant immune mechanisms in cancer. Nature. 2015;520:373–7.

16. Mosely SIS, Prime JE, Sainson RCA, Koopmann J-O, Wang DYQ, Greenawalt DM, et al. Rational Selection of Syngeneic Preclinical Tumor Models for Immunotherapeutic Drug Discovery. Cancer Immunol. Res. 2017;5:29–41.

17. Sharabi AB, Nirschl CJ, Kochel CM, Nirschl TR, Francica BJ, Velarde E, et al. Stereotactic Radiation Therapy Augments Antigen-Specific PD-1-Mediated Antitumor Immune Responses via Cross-Presentation of Tumor Antigen. Cancer Immunol. Res. 2015;3:345–55.

18. Brenner DJ, Hlatky LR, Hahnfeldt PJ, Huang Y, Sachs RK. The linear-quadratic model and most other common radiobiological models result in similar predictions of time-dose relationships. Radiat. Res. 1998;150:83–91.

19. Sachs RK, Hlatky LR, Hahnfeldt P. Simple ODE models of tumor growth and anti-angiogenic or radiation treatment. Math. Comput. Model. 2001;33:1297–305.

20. Watanabe Y, Dahlman EL, Leder KZ, Hui SK. A mathematical model of tumor growth and its response to single irradiation. Theor. Biol. Med. Model. [Internet]. 2016 [cited 2017 Mar 4];27;13:6. Available from: http://www.tbiomed.com/content/13/1/6

21. Williams MV, Denekamp J, Fowler JF. A review of alpha/beta ratios for experimental tumors: implications for clinical studies of altered fractionation. Int. J. Radiat. Oncol. Biol. Phys. 1985;11:87–96.

22. Gerritsen B, Pandit A. The memory of a killer T cell: models of CD8+ T cell differentiation. Immunol. Cell Biol. 2016;94:236–41.

23. Kaech SM, Cui W. Transcriptional control of effector and memory CD8+ T cell differentiation. Nat. Rev. Immunol. 2012;12:749–61.

24. Kim PS, Lee PP, Levy D. Modeling regulation mechanisms in the immune system. J. Theor. Biol. 2007;246:33–69.

25. Chen DS, Mellman I. Oncology Meets Immunology: The Cancer-Immunity Cycle. Immunity. 2013;39:1–10.

26. Selby MJ, Engelhardt JJ, Quigley M, Henning KA, Chen T, Srinivasan M, et al. Anti-CTLA-4 Antibodies of IgG2a Isotype Enhance Antitumor Activity through Reduction of Intratumoral Regulatory T Cells. Cancer Immunol. Res. 2013;1:32–42.

27. Chen L, Han X. Anti–PD-1/PD-L1 therapy of human cancer: past, present, and future. J. Clin. Invest. 2015;125:3384–91.

28. Filatenkov A, Baker J, Mueller AMS, Kenkel J, Ahn G-O, Dutt S, et al. Ablative Tumor Radiation Can Change the Tumor Immune Cell Microenvironment to Induce Durable Complete Remissions. Clin. Cancer Res. 2015;21:3727–39.

29. Azad A, Yin Lim S, D’Costa Z, Jones K, Diana A, Sansom OJ, et al. PD‐L1 blockade enhances response of pancreatic ductal adenocarcinoma to radiotherapy. EMBO Mol. Med. 2017;9:167–80.

30. Deng L, Liang H, Burnette B, Beckett M, Darga T, Weichselbaum RR, et al. Irradiation and anti–PD-L1 treatment synergistically promote antitumor immunity in mice. J. Clin. Invest. 2014;124:687–95.

31. Dovedi SJ, Melis MHM, Wilkinson RW, Adlard AL, Stratford IJ, Honeychurch J, et al. Systemic delivery of a TLR7 agonist in combination with radiation primes durable antitumor immune responses in mouse models of lymphoma. Blood. 2013;121:251–9.

32. Takeshima T, Chamoto K, Wakita D, Ohkuri T, Togashi Y, Shirato H, et al. Local Radiation Therapy Inhibits Tumor Growth through the Generation of Tumor-Specific CTL: Its Potentiation by Combination with Th1 Cell Therapy. Cancer Res. 2010;70:2697–706.

33. Zeng J, See AP, Phallen J, Jackson CM, Belcaid Z, Ruzevick J, et al. Anti-PD-1 Blockade and Stereotactic Radiation Produce Long-Term Survival in Mice With Intracranial Gliomas. Int. J. Radiat. Oncol. 2013;86:343–9.

34. Lee Y, Auh SL, Wang Y, Burnette B, Wang Y, Meng Y, et al. Therapeutic effects of ablative radiation on local tumor require CD8+ T cells: changing strategies for cancer treatment. Blood. 2009;114:589–95.

35. Deng L, Liang H, Burnette B, Beckett M, Darga T, Weichselbaum RR, et al. Irradiation and anti–PD-L1 treatment synergistically promote antitumor immunity in mice. J. Clin. Invest. 2014;124:687–95.

36. Park SS, Dong H, Liu X, Harrington SM, Krco CJ, Grams MP, et al. PD-1 Restrains Radiotherapy-Induced Abscopal Effect. Cancer Immunol. Res. 2015;3:610–9.

37. Topalian SL, Taube JM, Anders RA, Pardoll DM. Mechanism-driven biomarkers to guide immune checkpoint blockade in cancer therapy. Nat. Rev. Cancer. 2016;16:275–87.

**Legends for Supplemental Figures**

**Figure S1. Model diagnostic plots.** **(A)** Observations vs. population model predictions**.** **(B)** Observations vs individual model predictions. The straight line represents a perfect agreement between experimental and calculated values. **(C)** Distribution of the individual fitted values for parameter SL**. (D)** Population weighted residuals distribution. **(E)** Individual weighted residuals distribution. **(F)** Visual predictive check. Black arrows: RT administration (fractionated dose of 5 x 2 Gy); blue arrows: anti PD-L1 mAB administration (3qw for 3 weeks). Experimental data are taken from Dovedi et al. [1].

**Figure S2. Evaluation of model predictions against experimental data.** Tumor size dynamics data (black dots) population (red lines) and individual (green lines) model predictions **(A)** Control. **(B)** anti PD-L1 3qw on Day 7. **(C)** 5x2Gy RT. **(D)** 5x2Gy RT + anti PD-L1 3qw on Day 7. **(E)** 5x2Gy RT + anti PD-L1 3qw on Day 12. **(F)** 5x2Gy RT + anti PD-L1 3qw on Day 19.

**Figure S3. Evaluation of a common anti-tumor efficacy criterion, under various treatment scenarios.** In all treatments, RT was starting on Day 7 following tumor inoculation. A total of 2000 virtual experiments were simulated - each including 10 animals per cohort - for each treatment modality. *Per* treatment, median efficacy (% of responders, black solid line) with a 90% confidence interval (CI, black box) were calculated. Red circles: experimentally observed percentages of animals with anti-tumor efficacy [1,12], using the same criterion as defined in the model-based simulations.

**Figure S4. Model sensitivity analysis.** Sensitivity is represented as a percentage change in treatment efficacy (in this figure: 5x2Gy RT + anti PD-L1 3qw on Day 7), upon increasing or decreasing each parameter value, individually, by +/- 10% around a median value (only values for *kpro* and *kdif* were modified simultaneously, since these two parameters are strongly coupled given the model structure).

**Figure S5. Model simulations RT started on Day 19 after tumor cell injection. (A)** Efficacy simulation results, summarized as percentages of ‘responders’ (animals exhibiting full tumor rejection; defined as a total tumor volume ≤10 mm3 on Day 50 following treatment start), median with 90% CI (values in brackets), based on 1000 virtual studies with 100 animals per study. **(B)** Simulations of corresponding maximal levels of mature DCs (maximal value of *DCm* model variable). **(C)** Simulations of corresponding cytotoxic effector T cells (*dTeff* model variable).

**Table S1. Final model selection**

| **Model**  **#** | **Objective Function Value (OFV)FV** | **Immune system activation by TCD** | **PD-L1 effect on the immune system** | **Random effect** |
| --- | --- | --- | --- | --- |
| 1 | 4394 |  | Reduce Teff efficacy | kLN |
| 2 | 4397 |  | Suppress activation | kLN |
| 3 | 4416 |  | Reduce Teff efficacy | kLN |
| 4 | 4420 |  | Suppress activation | kLN |
| 5 | 4383 |  | Reduce Teff efficacy | kLN |
| 6 | 4388 |  | Suppress activation | kLN  ΩkLN=0.25 |
| 7 | 4318 |  | Suppress activation | sL  ΩkLN=0.95 |
| 8 | 4393 |  | Suppress activation | e  Ωe=0.27 |
| 9 | 4447 |  | Suppress activation | sR  ΩsR =0.65 |
| 10 | 4374 |  | Suppress activation | r  Ωr =0.10 |
| 11 | 4318 |  | Suppress activation | Ωr =0.05  +  ΩsL =1.32 |

**Table S2 (in separate excel file). Simulated values with 90% confidence intervals for efficacy, maximal mDC and dTeff levels.**
